# LLM-Assessed Relatedness of Microbiome Study Descriptions Aligns more Strongly with Functional than with Taxonomic Profile Similarity

**DOI:** 10.1007/s00248-026-02730-5

**Published:** 2026-03-31

**Authors:** Nefeli Kleopatra Venetsianou, Savvas Paragkamian, Konstantinos Kalaentzis, Alexios Loukas, Christina Damianou, Vincenzo Lagani, Lars Juhl Jensen, Evangelos Pafilis

**Affiliations:** 1 Institute of Marine Biology, Biotechnology and Aquaculture, Hellenic Centre for Marine Research, Crete, Greece; 2 Department of Biology, University of Crete, Heraklion, Crete Greece; 3 Hydrobiological Station of Rhodes, Hellenic Centre for Marine Research, Rhodes, Greece; 4 Institute of Marine Biological Resources and Inland Waters, Hellenic Centre for Marine Research, Crete, Greece; 5 Division of Biomedical Sciences, King Abdullah University of Science and Technology, Thuwal, Saudi Arabia; 6 Institute of Chemical Biology, Ilia State University, Tbilisi, Georgia; 7 Novo Nordisk Foundation Center for Protein Research, University of Copenhagen, Copenhagen, Denmark; 8 ZS Discovery, Kongens Lyngby, Denmark

## Abstract

Microbiome studies reveal the taxonomic and functional composition of microbial communities inhabiting many diverse environments. Comprehensive microbiome repositories, such as MGnify, organize data into studies, each consisting of multiple sequencing runs or assemblies and accompanying metadata. This structure enables integrative, large-scale, cross-study analyses, leading to broader insights across ecosystems, hosts, and experimental contexts. Despite extensive microbiome research, methods for defining similarity between studies and validating those similarity metrics, remain insufficiently established, especially for large-scale analyses. To address this, we evaluate whether taxonomic and functional similarities from MGnify can serve as reliable indicators of study relatedness between study pairs, testing multiple metrics against conceptual relatedness (e.g., shared environments, goals, or methods). To scale validation, we introduce a framework that applies a Large Language Model (LLM) to study descriptions, categorizing study pairs by relatedness. Our results show that functional similarity correlates more strongly with LLM-inferred study relatedness than taxonomic similarity, highlighting both the promise and limitations of current metrics. Via the above, we demonstrate the value of combining microbial profiles with LLM-driven semantic reasoning to navigate the expanding landscape of metagenomic research.

**Supplementary Information:**

The online version contains supplementary material available at 10.1007/s00248-026-02730-5.

## Introduction

A fundamental challenge in microbiome research is understanding how different studies relate to each other, specifically how the taxonomic and functional similarity of microbiome studies correlates with their broader environmental or scientific relatedness [1]. The availability of large centralized databases such as MGnify [2], JGI IMG/M [3], and SPIRE [4] has enabled access to thousands of samples and studies encompassing global microbiome data. Each study (or project) can have multiple samples, which contain raw sequences accompanied by metadata checklists [5], taxonomic and functional profiles [6]. Hence, these resources allow for global meta-analyses of microbiomes. 

Meta-analyses can be implemented either by integrating samples from multiple studies into a single unified dataset or by comparing studies based on their respective sample sets, which preserves study distinctions. In the latter approach, samples are analyzed within each study separately, and the results are then aggregated or summarized at the study level to enable comparisons across studies. This preserves differences and similarities in study design and sampling while allowing meta-analysis at the study level. The former approach has led to the identification of important microbiome drivers in the ocean [7] and soil [8], and to new insights about environmental niches and strategies of microbes [9]. However, with the latter approach, many questions arise due to the large number of studies and their unstructured descriptions. How to define and measure similarity, and which similarity metrics best capture meaningful biological relationships? Can such approaches scale across thousands of studies and their samples, and, importantly, how can we validate the biological similarity of studies in a consistent and scalable manner? Addressing these questions is crucial for enhancing analytical approaches, facilitating study clustering, and supporting knowledge discovery across microbiome datasets.

The similarity between microbial communities (samples β diversity) is commonly calculated using the Jaccard index [10], for presence/absence-based data, Bray-Curtis dissimilarity and Jensen–Shannon divergence [11] for compositional data (relative abundances, read counts) [12]. To satisfy the need for a more accurate microbial community similarity index, researchers are developing new methods and tools to incorporate phylogenetic information (i.e. UniFrac [13]), functional redundancy (i.e PhyloFunc [14]), microbial interactions (i.e PINA [12]) and more sophisticated statistical calculations (i.e. FAVA [15]). Still, the scalability of these methods remains a challenge when applied to large-scale datasets involving thousands of samples across studies, highlighting the need for computationally efficient, interpretable, and generalizable similarity measures. Recent tools such as Libra, address scalability by enabling efficient all versus all similarity calculations directly from raw metagenomic sequences, bypassing the need for taxonomic or functional annotations [16].

Metagenomic studies are singular objects in databases that frame the research questions and experimental design, while linking sample sequence data with metadata [2]. It is an open question whether the similarity of taxonomic or functional profiles of samples correlates with the relatedness of studies in a broader, conceptual sense. However, studies might display highly similar microbial compositions yet not be thematically related. For example, two ocean microbiome studies may include samples with similar community composition yet differ in terms of scientific goals, methodologies, or experimental designs [17]. This complicates efforts to group similar studies and to identify transferable insights across studies. Consequently, quantifying relatedness between studies requires an integrated analysis of biological similarity (taxonomic/functional) and semantic or contextual similarity (e.g., metadata, study descriptions, research questions).

Traditionally, assessing whether two studies are related has been a manual process, relying heavily on expert judgement of study descriptions and other metadata [17]. While this approach ensures high accuracy and domain awareness, it does not scale to the thousands of studies now available in repositories, particularly considering that more are constantly added. Moreover, consistency in human judgment can vary, and even expert assessments will differ due to the subjective nature of interpreting what constitutes relatedness [5, 18].

Large Language Models (LLMs) offer a compelling alternative to manual annotation, as they can analyze unstructured text such as study abstracts, descriptions, and other metadata at scale [19]. In the context of microbiome research, Daniela Gaio et al. [20] have utilized LLMs to process textual metadata from sequencing records in the environment and categorize these records into broad ecological environments. In addition, with appropriate guidance in the form of prompt engineering, LLMs can extract and interpret latent semantic relationships, which enables automated assessment of conceptual relatedness between studies [21]. Advances in the area highlight the potential of prompt engineering to enhance reasoning, automate research workflows, and improve the consistency and reliability of LLM outputs [22, 23] thereby facilitating more efficient discovery and integration of knowledge across microbiome studies.

In this study, we explore the taxonomic and functional similarity metrics derived from microbiome studies in MGnify. We aim to decipher the scientific relatedness of studies, as inferred from textual study descriptions combined with their similarity taxonomy and functional profiles. To this end, we developed a scalable framework that leverages an LLM to semantically compare study descriptions and assign relatedness levels. We further investigated which similarity profile best correlates with the semantic relatedness between studies inferred by the LLM. The similarity metrics we chose (Cosine similarity, Euclidean distance and Jensen–Shannon divergence), were selected because they capture complementary aspects of microbiome profile similarity while remaining computationally efficient for large-scale analyses. This framework enables the validation of biological similarity scores through a complementary semantic lens, addressing the limitations of relying solely on taxonomic and functional profiles.

## Results & Discussion

### Evaluating the robustness of (dis)similarity metrics for metagenomics studies

We chose three widely used (dis)similarity metrics for our analyses: Euclidean distance, cosine similarity, and Jensen–Shannon divergence. To apply these numerical metrics to metagenomics samples and studies, we first projected each sample onto fixed-length numerical vectors. We use two distinct encodings: functional and taxonomical. In the taxonomical one, the vectors represent the relative abundances of a fixed set of genus-level organisms, as they are detected within the samples (see Methods for details). In the functional one, each element of the vector represents the proportion of reads assigned to a specific Gene Ontology (GO) term. The (dis)similarity between each pair of samples can now be easily computed by applying the chosen metrics on the corresponding numerical vectors. To compute the (dis)similarity between two studies, say study A and study B, which possibly contain different numbers of samples, we use a nearest neighbor approach. We first compute the (dis)similarity between each sample from study A and each sample from study B, and then we identify the maximum similarity (from A to B and vice versa). The final study-level similarity is defined as the maximum of the two directional averages, emphasizing the strongest cross-study correspondence.

To assess the robustness of the chosen similarity metrics, we introduced noise through multinomial downsampling of the count data, which approximately preserves the relative abundances. Noise injection in our framework is implemented through multinomial downsampling, which simulated stochastic variability arising specifically from differences in sequencing depth across studies. While experimental variability can arise from many factors, including protocols, extraction methods and sequencing platforms, our noise model is intentionally focused on depth-related sampling noise, as it is the most quantifiable and directly reproducible source of variability in metagenomic data [23–25]. A range of downsampling ratios (i.e., 0.1, 0.25, 0.75, 0.9) was used on a test set of 4,000 samples for taxonomic data and 2,000 samples for functional, introducing controlled probabilistic variation to simulate reduced sequencing depth. The objective of this analysis is to identify the metric that best manages to assign the noisy samples to their corresponding original samples.

**Fig. 1:**
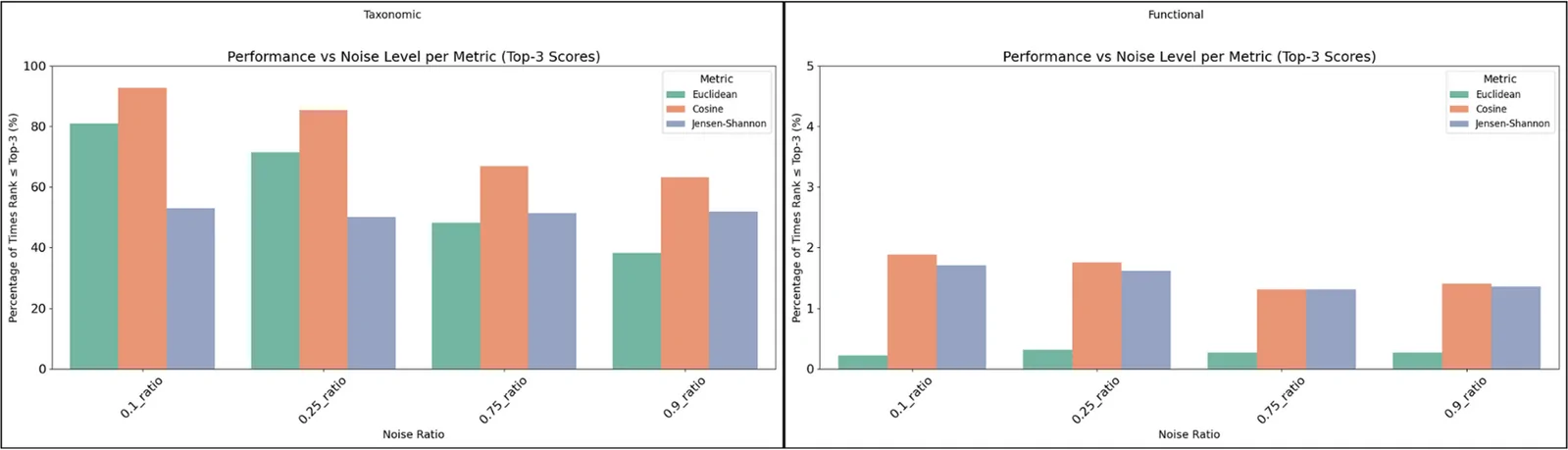
Top-3 retrieval performance across noise levels for taxonomic and functional profiles. The plots show the percentage of times the original sample was correctly ranked within the top-3 most similar profiles after noise injection at varying levels (0.1, 0.25, 0.75, and 0.9) based on three metrics: cosine similarity (orange bar), Euclidean distance (green bar), and Jensen–Shannon divergence (purple bar). (Left: Taxonomic) Cosine similarity consistently outperforms Euclidean distance and Jensen–Shannon divergence, maintaining the highest match rate across all noise levels. Euclidean shows the steepest performance drop, while Jensen–Shannon remains relatively stable. (Right: Functional) All metrics exhibit closer performance. Cosine and Jensen–Shannon show similar resilience to noise, whereas Euclidean remains consistently lower. These trends confirm cosine as the most robust metric, particularly for taxonomic data

Each noisy sample was compared against all original profiles, and the rank of the correct match was recorded. Performance was evaluated at multiple top-k levels (Top-1, Top-3, Top-5, Top-10, Top-100), capturing how often the correct match appeared among the most similar profiles. As shown in Fig. 1, cosine similarity shows a clear tendency to outperform the other two metrics at all noise levels for both taxonomic and functional profiles. As noise increased, performance declined across all metrics; however, cosine similarity retained a clear advantage in the taxonomic data. These results highlight cosine similarity as the most robust metric for preserving sample identity even under high noise levels, in line with previous work [16]. Results for the rest of the top-k ranks (i.e., top-1, top-5, top-10, top-100) can be found in the “Supplementary Material: 1. Noise Injection” for both taxonomic and functional data.

Functional abundance tables are typically more sparse than taxonomic profiles, and the abundance per feature is often considerably lower. This sparsity leads to weaker signal-to-noise ratios and can cause similarity metrics to behave less reliably under perturbation. Accordingly, functional similarity scores tend to be lower and more variable, especially when noise is introduced or when the comparison involves Top-k matching.

Given these results, we decided to use cosine similarity for all subsequent sample comparisons. By aggregating sample-to-sample similarities at the study level, we identified studies with similar taxonomic or functional profiles.

### Study-Level Relatedness Based on Text via Large Language Models (LLM)

**Fig. 2:**
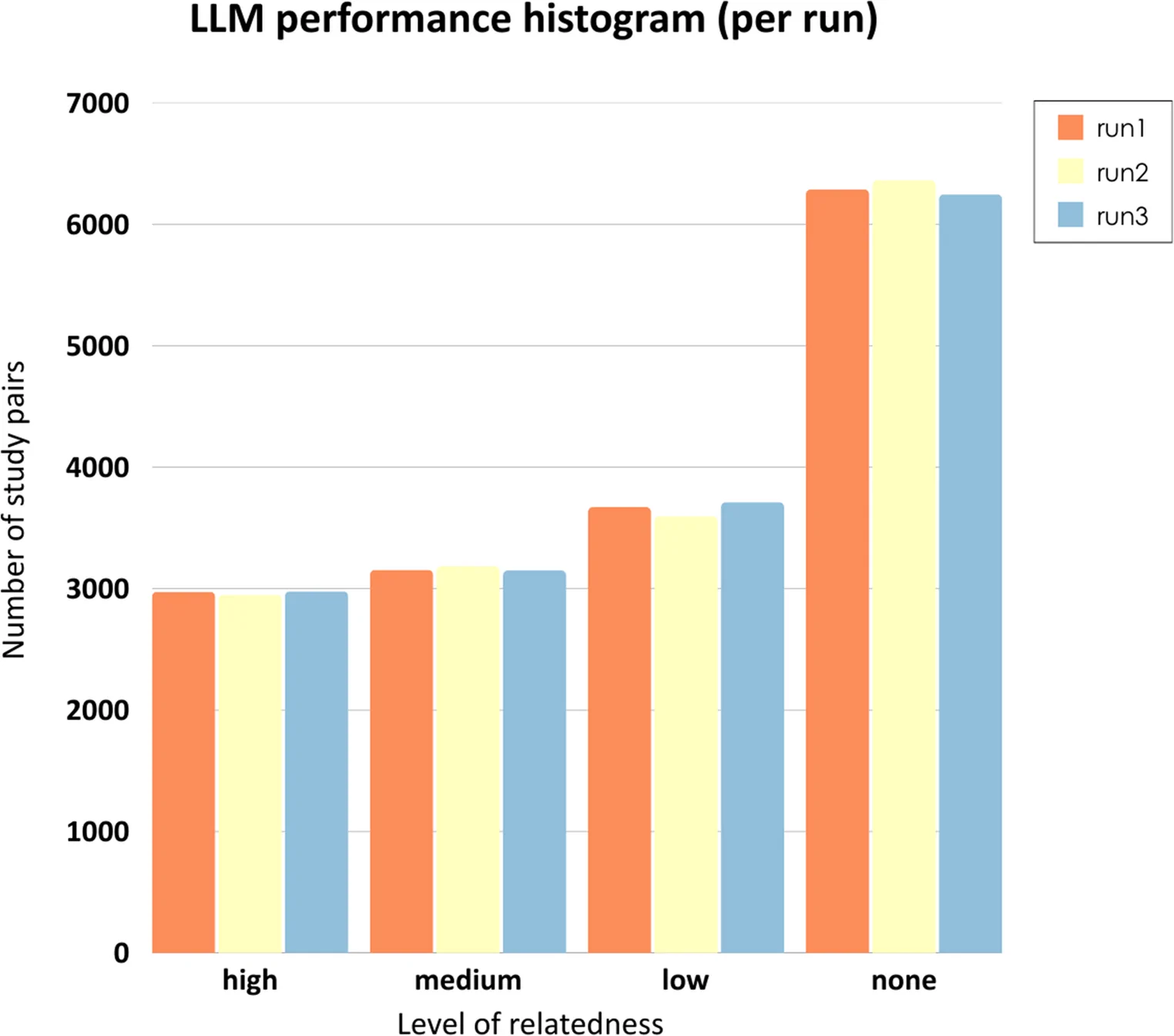
LLM similarity classification consistency across runs. LLM performance histogram showing the number of study pairs classified into four similarity categories (“high”, “medium”, “low”, and “none”) across three independent runs (run1, run2, run3). Results indicate consistent distribution patterns, highlighting the model's robustness in evaluating study-level textual similarity

To capture conceptual relatedness between studies, the similarity of each study pair was explored using a large language model (LLM, see Methods for details). The LLM analyzed the textual content of each study pair and categorized their relatedness into four levels: “none,” “low,” “medium,” and “high.” In total, we proceeded with 16,194 study pairs, selected based on the availability of taxonomic or functional profiles, to enable cross-study comparisons. In addition, the LLM was executed three independent times to assess inference variability and output stochasticity. The results, as shown in the histogram in Fig. 2, revealed a consistent distribution across all runs, with the majority of study pairs falling into the “none” category, indicating no apparent similarity. A smaller but stable number of pairs were classified as “low,” “medium,” and “high” across runs, reflecting a consistent pattern in the LLM's evaluation of relatedness. These findings demonstrate that the model’s similarity assessments are stable across repeated runs, supporting the reliability of the LLM’s output in terms of run-to-run consistency. While stability does not ensure correctness, it provides a necessary foundation for using the LLM as a robust tool for assessing textual relatedness in scientific literature [26].

To statistically assess whether taxonomic and functional similarity distributions significantly differed across the four LLM-relatedness categories (“none,” “low,” “medium,” “high”), we first applied the Kruskal-Wallis test. This provided an overall test of differences among categories. This test yielded highly significant results for both similarity types (Functional and Taxonomic) across all three runs (Taxonomic: H-statistic 80.64–107.68.64.68, p < 1e-16; Functional: H-statistic = 380.5–457.7, p < 1e-80), indicating that at least one category distribution differed from all the others. Following this, we conducted pairwise Mann-Whitney U tests to identify which configurations in particular differed from the others.

For each similarity type (Functional and Taxonomic), three Mann–Whitney U tests were performed per run, comparing each LLM-relatedness category (“high”, “medium”, “low”) against the combined set of the remaining categories (i.e. high VS medium & low, medium VS high & low, low VS high & medium). The “none” category was excluded from these post-hoc comparisons because it contains substantially more studies than the other categories, and therefore could dominate the similarity distributions when pooled with the rest. This resulted in nine tests per similarity type across the three LLM runs (18 tests in total). P-values were corrected for multiple comparisons using the Benjamini–Hochberg FDR procedure [27, 28]. Across all runs, all adjusted p-values were < 0.05, supporting rejection of the null hypothesis (H₀) that the distributions are identical across LLM-relatedness categories. These results confirm that the similarity distributions differ significantly across relatedness levels for both functional and taxonomic similarity metrics. These results further demonstrate that LLM-derived textual similarity classifications correspond meaningfully, yet differently, with structured similarity metrics, depending on the metadata type and LLM run. Detailed results of the post-hoc Mann-Whitney U tests are provided in the “Supplementary Material: 2. Summary of Mann-Whitney U Tests by LLM-Relatedness (Table S1)”.

### LLM-Derived Semantic Similarity Aligns Best with Functional Similarity

Further examination of taxonomic and functional profile similarity distribution across LLM-relatedness levels provided nuanced insights. For study pairs categorized as having no LLM-relatedness, the distribution of similarities was relatively broad, with a notable peak at very high similarities (close to 1), followed by a gradual tapering across lower similarities. This suggests that even among unrelated studies, some pairs exhibit taxonomic or functional similarities, albeit more diffusely spread.

**Fig. 3:**
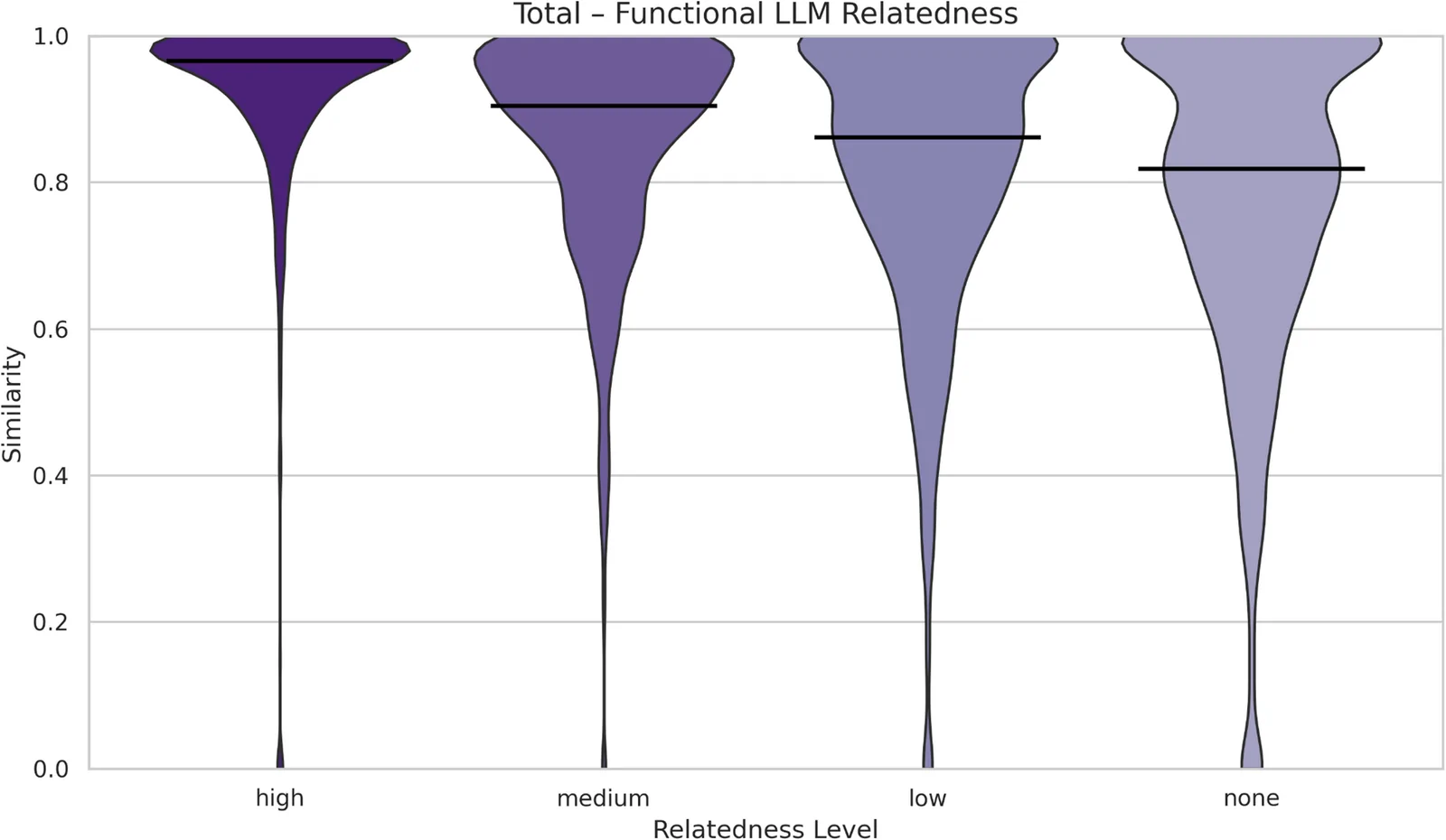
Violin plots of functional similarity across study pairs, grouped by LLM-assessed semantic relatedness. The x-axis represents LLM-assessed semantic relatedness across four levels (high, medium, low, none), while the y-axis shows the functional cosine similarity metric (range 0–1), where higher values indicate greater functional overlap. The width of each violin reflects how frequently similarity values occur at a given level, and the horizontal bar indicates the median similarity within each group. The figure illustrates how the spread and central tendency of functional similarity vary across levels of semantic relatedness

Further analysis of the distributions of functional profile similarities across different levels of LLM-relatedness revealed clear and consistent trends (Fig. 3). In the “none” relatedness category, the distribution was broad and right-skewed, with a prominent initial spike at very high similarity scores followed by a long tail. This indicates that while the LLM deemed these study pairs to have no textual similarity, a subset still exhibited high functional similarities, which may reflect underlying ecological or biological similarity from the study descriptions, rather than purely incidental overlaps. For study pairs classified as having “low” and “medium” relatedness, the distribution remained skewed toward higher similarities but was more concentrated than the “none” category. A substantial portion of pairs clustered above 0.6, suggesting that even limited textual similarity often exhibited notable functional similarity. This indicates a weak but noticeable alignment between LLM-derived and functional similarity distance, potentially reflecting broad baseline similarities across specific biome samples that persist even when studies have little else in common. The “high” relatedness category displayed a markedly different pattern. Here, the vast majority of functional profile similarities were extremely high, predominantly above 0.9. This tighter distribution signifies a strong correspondence between the LLM’s semantic similarity assessments and functional alignment, highlighting the model’s effectiveness in capturing meaningful relatedness between studies. Overall, these distributions reveal a monotonic relationship; as LLM-rated relatedness increases, the functional similarity increases and becomes more tightly concentrated around one. This pattern not only supports the internal consistency of the LLM's categorization but also provides external validation, through functional similarity metrics, of the model's capacity to detect substantively related study pairs. This affirms the value of LLMs in augmenting literature analysis by providing reliable and scalable assessments of study-level similarity.

**Fig. 4:**
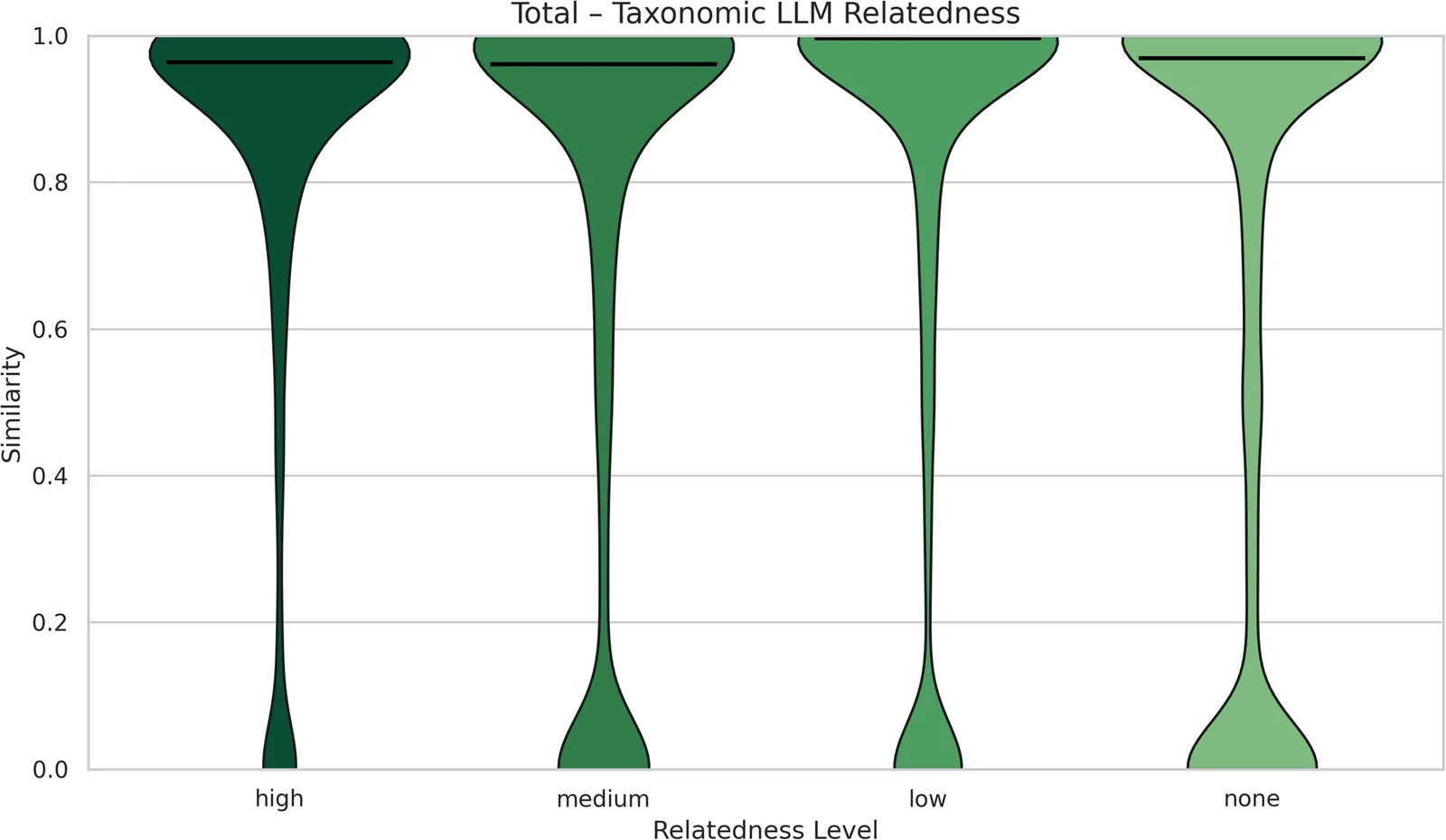
Violin plots of taxonomic similarity across study pairs, grouped by LLM-assessed semantic relatedness. The x-axis represents LLM-assessed semantic relatedness across four levels (high, medium, low, none), while the y-axis shows the taxonomic cosine similarity metric (range 0–1), where higher values indicate greater taxonomic overlap. For each category, the width of the violin reflects how frequently similarity values occur at a given level, and the horizontal bar denotes the median similarity. Across all relatedness levels, similarity values are concentrated toward the upper end of the range, with differences among categories primarily reflected in the spread of the distributions and the presence of low-similarity tails

Notably, a different pattern emerged in the taxonomic profile similarity analyses (Fig. 4). Across all LLM-relaredness categories, taxonomic similarity values are strongly skewed toward the upper end of the distribution, indicating that high taxonomic overlap is common regardless of semantic relatedness. Differences among categories are insted reflected in the spread of the distributions and the presence of low-similarity components. In particular, the “medium” and “none” relatedness groups show more pronounced low-similarity tails, whereas the “high” relatedness group exhibits a comparatively narrower distribution with minimal density near zero. This contrasts with functional similarity rewsults, where low-similarity modes are less prominent, suggesting that functional similarity remains

This counterintuitive result suggests a potential disconnect between semantic similarity and taxonomic overlap in the current structure. Several factors may explain this phenomenon. Firstly, taxonomic metadata may be too sparse or inconsistently applied across studies, reducing the signal available for meaningful comparison. Unlike functional annotation, which often follows standardized vocabularies and pipelines, taxonomic metadata may depend heavily on variable factors such as sampling methodology, resolution (e.g., genus vs. species level), or reporting practices. Moreover, inverse correlations can arise when different taxonomic groups occupy similar ecological niches or fulfill comparable roles within ecosystems [29]. In such cases, communities that appear highly divergent at the taxonomic level may nevertheless share broad ecological characteristics, leading to situations where lower taxonomic similarity coincides with stronger semantic similarity.

Taken together, this suggests that taxonomic similarity may be more vulnerable to noise and inconsistencies in metadata than functional similarity, potentially limiting its reliability as a standalone measure of study-relatedness. These insights underscore the importance of improving metadata quality, and refining similarity algorithms. 

**Fig. 5:**
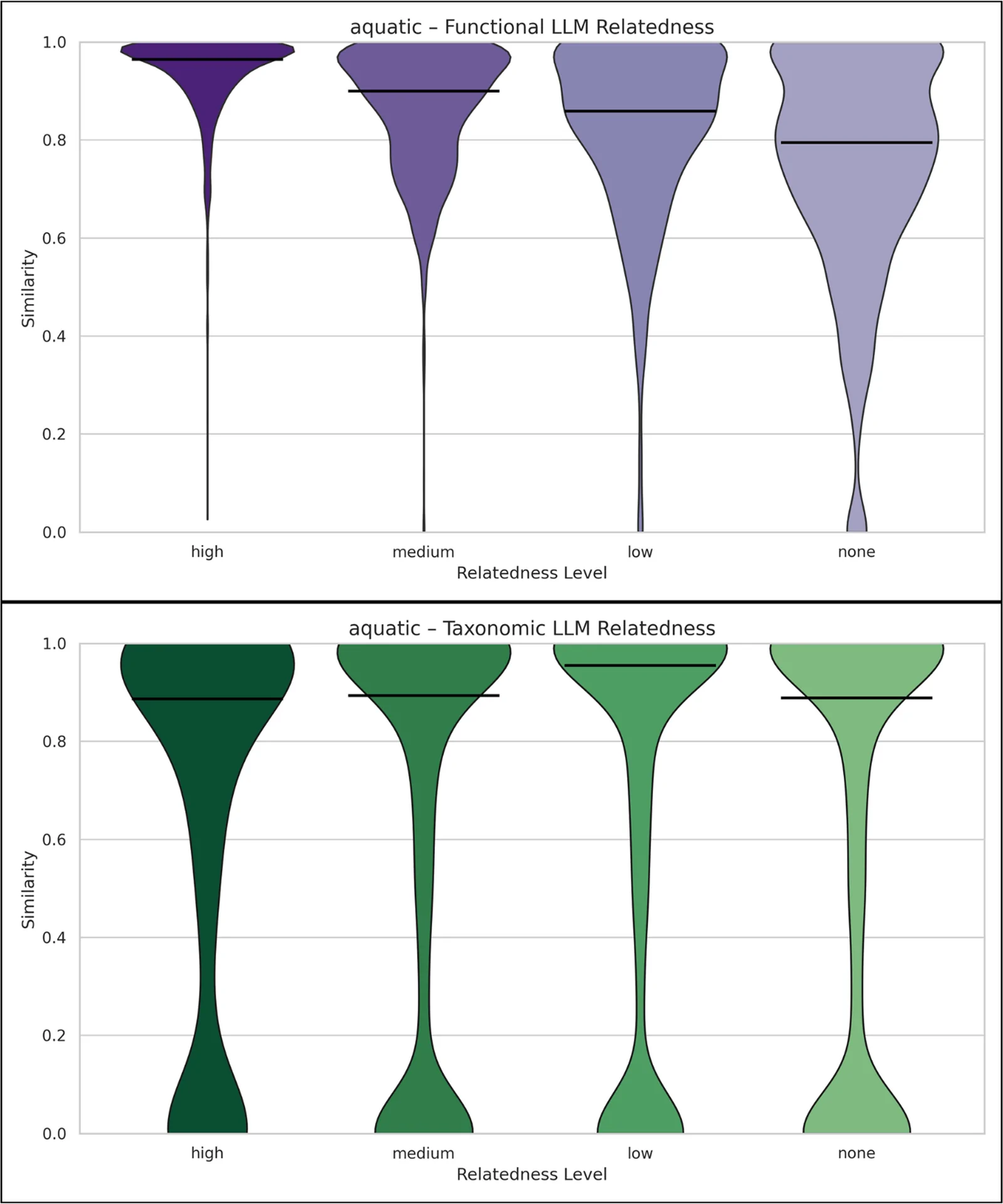
**Violin plots of functional (a. purple violin plots) or taxonomic (b. green violin plots) similarity for aquatic study pairs across biomes, stratified by LLM-assessed semantic relatedness .**The x-axis shows LLM-assessed semantic relatedness levels: high, medium, low, none. The y-axis shows functional similarity values (range 0–1), where higher values indicate greater functional overlap. The width of each violin reflects the frequency of similarity values at a given level, and the horizontal bar indicates the median. **a. Functional:** Across categories, the distributions show consistently high similarity, with the medium and high relatedness groups exhibiting dense concentrations near maximum similarity, indicating strong functional alignment for semantically related studies. The low and none categories also display substantial density near high similarity, revealing that some semantically distant pairs still appear functionally similar, potentially reflecting broader shared ecological processes or false-positive matches. Nonetheless, it is clear that as LLM relatedness decreases, the density shifts downward, highlighting a monotonic relationship between semantic and functional similarity. **b.Taxonomic:**While taxonomically related studies show the expected high-density regions near maximum similarity, the none and low relatedness groups also contain substantial density at high similarity values. This pattern suggests that many semantically unrelated aquatic studies nevertheless share considerable taxonomic overlap. The broad and sometimes bimodal distributions further indicate that the taxonomic algorithm may emphasize higher-level or widespread aquatic taxa rather than fine-grained species distinctions, leading to elevated similarity even among semantically distant study pairs

**Fig. 6:**
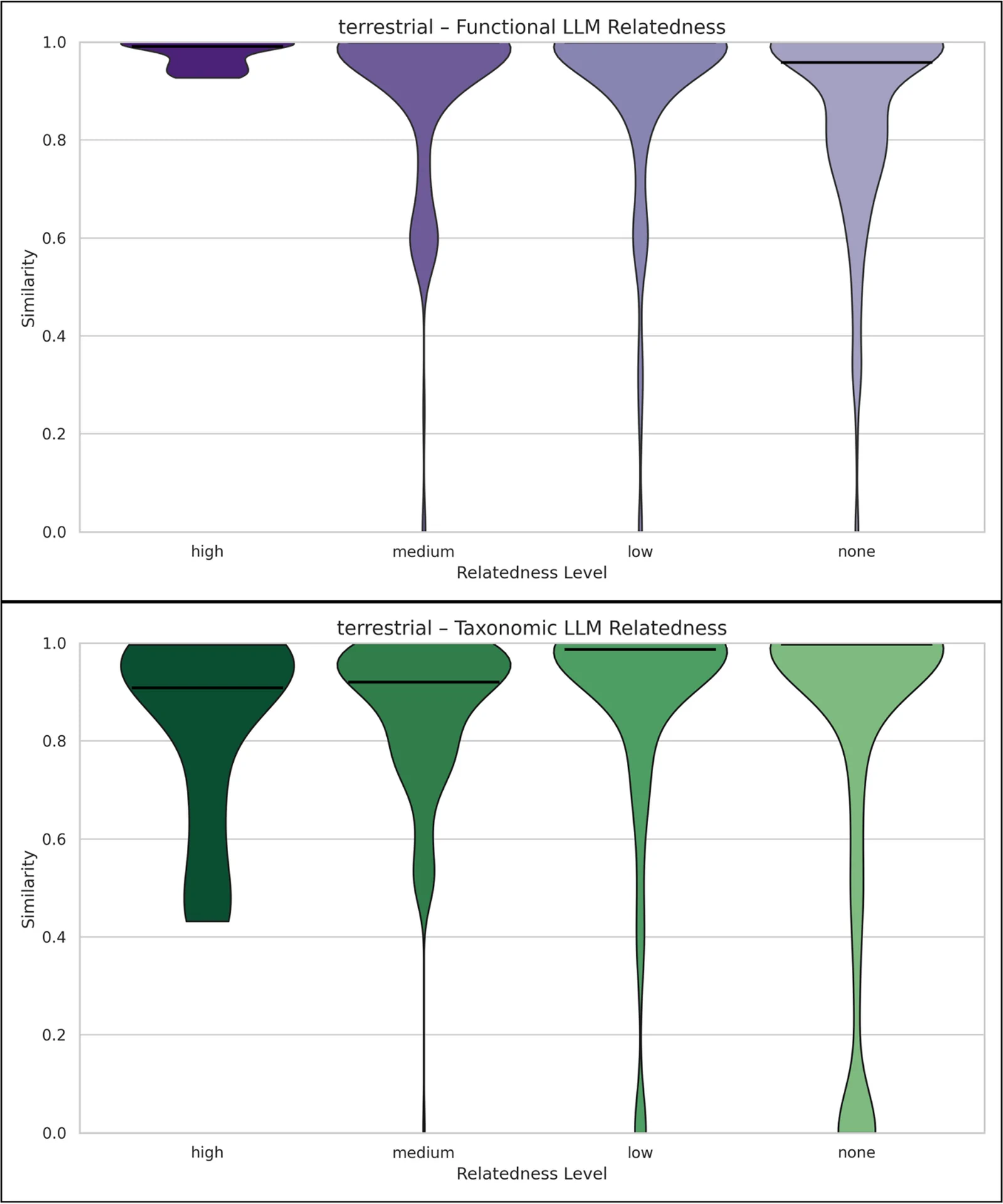
**a**. Violin plots of functional (purple violin plots) or taxonomic (green violin plots) similarity for terrestrial study pairs across biomes, grouped by LLM-assessed semantic relatedness. The x-axis shows LLM-assessed semantic relatedness levels: high, medium, low, none. The y-axis shows functional similarity values (range 0–1), where higher values indicate greater functional overlap. The width of each violin reflects the frequency of similarity values at a given level, and the horizontal bar indicates the median. a. Functional: Highly related pairs show dense distributions near maximum similarity, while medium-related pairs exhibit a mojority of values up to 0.6, indicating strong alignment between semantic and functional similarity. As LLM relatedness decreases, the distribution shifts downward, reflecting a monotonic trend in which lower semantic relatedness corresponds to lower functional similarity. Nonetheless, the low and none categories still show nontrivial density near high similarity, suggesting either broad terrestrial functional convergence or occasional false-positive matches. b. Taxonomic: Although many highly and moderately related pairs exhibit strong concentrations near high taxonomic similarity, the none and low relatedness categories also show substantial density at very high similarity values. This pattern suggests that taxonomic overlap remains widespread even among semantically unrelated studies, likely reflecting high-level taxonomic similarity within terrestrial biomes that limits fine-scale resolution. The broader and occasionally bimodal shapes of these distributions highlight that semantic relatedness is not a reliable predictor of taxonomic alignment for terrestrial studies

To further assess whether the observed disconnect between semantic and data-driven similarity holds across different ecological contexts, we conducted a stratified analysis by biome. Specifically, we examined results across four representative biomes, i.e., mammals, plants, aquatic, and terrestrial, and four extreme environments, i.e., contaminated, mines, volcanic, and deep sea. This biome-level slicing revealed an additional strength of the study matcher algorithm (Figs. 5, 6, “Supplementary Material: 3. LLM tested per biome” - Figures 11–12): for cases of medium to high relatedness, the study-matcher similarity algorithm tended to exhibit predominantly high similarity values,, particularly in terms of functional profiles. This suggests that the algorithm is functioning effectively in identifying genuinely related studies. However, in cases with low or no apparent LLM-relatedness, the algorithm frequently assigned very high similarity in terms of both functional and taxonomic profiles. Rather than necessarily indicating false positives, this pattern reflects the baseline functional and taxonomic similarity that persists within certain ecological contexts. For example, many aquatic samples share broad community structures and ecological signatures, so even unrelated studies may appear similar at the level of functional or taxonomic profiles [29]. Because the study-matcher algorithm captures similarity across these high-level taxonomic and functional features, even studies without direct experimental overlap can produce high similarity scores. Comparable effects may also occur in other biomes with strong, large-scale, biome-wide signatures, such as terrestrial soils [30]. In such cases, studies might not overlap in fine-grained detail but still exhibit general thematic or structural resemblance, which the algorithm interprets as relatedness. An example is provided below in Fig. 5a for functional results and 5b for taxonomic results of aquatic data, while Figs. 6a and 6b contain the functional and taxonomic results of terrestrial data, respectively. Results for additional biomes can be found in the “Supplementary Material: 3. LLMs tested per biome” (Figures 12–13).

Finally, a notable difference in the taxonomic similarity plots of Figs. 5 and 6 (for all levels of LLM relatedness) is the bimodal shape observed in the aquatic study pairs (Fig. 5), as opposed to the dense high taxonomic similarity region in the terrestrial ones (Fig 6). As community composition is a robust habitat predictor [31], such pattern might arise because terrestrial MGnify studies are dominated by soil ones, while aquatic studies are mostly marine, some freshwater ones, as well as others. This bimodality in taxonomic similarity is consistent with a well-established ecological phenomenon: communities tend to cluster either as highly similar or entirely distinct, depending on their environmental context. In contrast, the functional similarity distributions are unimodal, reflecting the functional redundancy commonly observed in microbial ecosystems, where different taxa can perform similar functions across diverse habitats [29, 32].

### Examining Contradictory Results of Functional/Taxonomic Calculated Similarity Versus LLM Relatedness

In the subset of 20 studies we examined, the relationship between functional or taxonomic similarity and LLM-based relatedness occasionally appeared contradictory. For taxonomic similarity, we often observed high similarity even when LLM-based relatedness was low or absent. A likely explanation is that many studies are dominated by a small number of highly abundant taxa. When a few dominant taxa account for most of the taxonomic signal within a study, two studies can appear artificially similar even though their overall community compositions differ substantially. This reflects inherent biological structure rather than a methodological issue, and it largely explains the **bimodal** pattern we observe in taxonomic similarity. Additionally, functional redundancy may contribute to the pattern in the “high” level of relatedness; even when taxonomic similarity is moderate, multiple taxa can perform similar ecological roles, leading to higher functional similarity, which was also observed in aquatic and terrestrial biomes.

Similarly, cases of high functional similarity but low or no LLM-based relatedness can arise for two main reasons. First, distinct taxa may encode genes that converge on similar functional properties, leading to similar functional profiles despite phylogenetic differences. However, this biological convergence does not necessarily translate into high LLM-based relatedness, which reflects the semantic similarity of the study descriptions; thus, even studies with similar profiles, may appear unrelated to the LLM when framed in different scientific contexts. Second, methodological factors, such as the use of Third-Party Annotation (TPA) pipelines for whole-genome shotgun (WGS) datasets, can homogenize functional annotations across studies. When comparable assembly and annotation workflows are employed, functional categories tend to be identified consistently, thereby increasing apparent functional similarity.

In a few specific cases, environmental and physiological constraints can also drive functional convergence despite taxonomic divergence. For example, the lime-injection acid sulfate soil study and the haloarchaea enzyme-production study investigate microbial communities exposed to strong chemical stress and extreme geochemical conditions. Although these ecosystems (grassland soil vs. hypersaline alkaline water) host very different taxa, both environments require microbes to deploy specialized functional strategies, including pH-adaptation mechanisms, stress-response pathways, ion-transport systems, homeostasis regulation, metabolic flexibility under extreme conditions, and production of enzymes adapted to harsh chemical environments. Such functional redundancy and convergence have been documented in microbial ecology, where distinct taxa maintain similar functional gene profiles under harsh environmental conditions, indicating overlapping ecological roles despite taxonomic differences [33]. Models and empirical analyses also show that environmental and thermodynamic constraints can cause microbial communities assembled from different species pools to converge in metabolic network structure and community function, independent of species composition [34]. This functional convergence can therefore produce high functional similarity even when LLM-based relatedness is low.

## Conclusions

Overall, our findings reveal a key pattern in the relationship between LLM-based relatedness and taxonomic/functional profile similarity metrics: LLM-derived relatedness scores align most strongly with functional similarity, with higher semantic relatedness consistently corresponding to higher functional similarity. The smooth and continuous transitions observed in functional similarity distributions, from closely related to unrelated pairs, further indicate the strong discriminative power of these embeddings across varying levels of biological similarity. While taxonomic similarity occasionally exhibits bimodal distributions in certain biomes, functional similarity provides a more robust and consistent measure. The bimodal behavior observed in taxonomic similarity can be largely driven by studies dominated by a few highly abundant taxa, which can create artificially high similarity despite substantial differences in overall community composition. These results also highlight the importance of carefully considering the context in which LLM outputs are interpreted in conjunction with structured metadata-based metrics. In line with ecological principles, our study matcher results on functional profiles similarity are consistent with the concept of functional redundancy as described by Louca et al. (2018) [35], whereby distinct taxonomic communities can maintain similar functional roles within microbial systems. This indicated that even when study texts are semantically unrelated their functional profiles may remain similar, while taxonomic composition may differ substantially. Finally, curating AI-ready metadata, structured, standardized, and rich in textual information, would further enhance such analyses, enabling accurate and nuanced exploration of functional and taxonomic profiles, potentially uncovering subtler patterns and relationships. 

Building on these findings, a key strength of the proposed framework lies in its ability to integrate LLM-derived semantic relatedness based on text with quantitative taxonomic and functional similarity metrics. This approach enables systematic validation of study-level similarity across thousands of microbiome studies, overcoming the scalability limitations of manual curation. Moreover, the observed monotonic relationship between LLM-based relatedness and functional profile similarity, provides biologically meaningful validation of the semantic judgements. Functional similarity shows smooth, continuous transitions across relatedness categories, suggesting that functional embeddings are particularly well suited for capturing meaningful overlap across studies, even when taxonomic composition differs substantially. Another strength of the framework is the robustness of the similarity metric selection. Through controlled noise injection, cosine similarity was shown to be resilient to sequencing depth variability, supporting its suitability for large-scale, heterogeneous metagenomic datasets.

At the same time, there are limitations of the methodology that should be acknowledged. Firstly, the aggregation of sample-level similarities into a single study-level score, might introduce inherent bias. The nearest-neighbor aggregation strategy emphasizes the strongest cross-study similarities, which is advantageous for detecting overlap, but may overestimate relatedness in cases where only a small subset of samples exhibits strong similarity while the remainder might diverge. In addition, functional similarity is influenced by annotation pipelines, which may inflate functional similarity scores independently of true ecological convergence. Lastly, taxonomic similarity is constrained by dominant taxa, a limitation that reflects both biological reality and methodological sensitivity, underscoring the need to interpret taxonomic similarity in conjunction with functional and semantic context.

In practice, to ensure that the workflow is applied properly, quality checks are recommended. Prior to downstream analysis, users should assess the completeness and quality of both textual metadata and compositional profiles. Studies with minimal or generic descriptions may yield unreliable LLM-based relatedness scores, and should be flagged for manual inspection. Similarly, users should verify basic properties of taxonomic and functional profiles, such as sequencing depth, or the dominance of a small number of taxa. Extreme dominance by a few taxa or unusually sparse functional profiles, can artificially inflate similarity scores and should therefore be interpreted with caution.

The interpretation of conflicting signals between semantic and compositional similarity can be challenging; however, such cases do not necessarily indicate errors, but often reflect meaningful biological or contextual distinctions. High functional similarity combined with low LLM-relatedness may reflect functional convergence across distinct ecosystems or experiment contexts, whereas high LLM-relatedness paired with low taxonomic or functional similarity may indicate differences in sampling strategies or experimental design, rather than true biological divergence.

Looking forward, appropriate follow-up validation analyses include inspection of sample-level similarity, stratification of biome, host or environmental variables (where available). This inspection will help to assess whether similarity is driven by broad contextual effects, exploration of alternative aggregation strategies or other similarity metrics might also be helpful to evaluate results, and finally targeted examination of key functional or taxonomic categories driving the observed similarity.

In conclusion, the proposed framework is best used as an exploratory and supplementary prioritization tool, guiding researchers toward candidate study pairs and highlighting cases of convergence or divergence that worth deeper investigation. By explicitly combining semantic, functional, and taxonomic perspectives, the approach encourages cautious interpretation and supports the identification of useful insights across studies, providing thus a solid foundation for more targeted validation and downstream meta-analyses.

## Materials & Methods

### Data Sources and Initial Processing

Both taxonomic and functional profiling data were obtained from MGnify [2], a centralized public platform providing standardized, preprocessed metagenomic data through consistent computational pipelines. MGnify's datasets span multiple microbiome biomes, including aquatic and terrestrial environments (marine, human gut, soil, etc.). A study represents a coherent metagenomic investigation defined by a research question and experimental design. Each study contains multiple runs (raw sequencing data) or in some cases assemblies (“environmental sample where Whole Genome Shotgun sequencing reads have been assembled to form larger fragments called contigs”) [6]. This database offers several versions for the different pipelines used to analyse a microbiome study's sequences (see “Supplementary Material: 4. MGnify Version-Pipeline description”). For this research project, we focused on MGnify versions v4.1 and v5.0 as they represent the latest, most refined and updated pipelines for downstream analyses. MGnify data was retrieved via MGnify’s API (as of March 4th, 2024). For each microbiome study, textual information, including title, description, and biome, was retrieved, along with the pertinent MGnify analysis data. Additionally, whenever a MGnify study was linked to related publications, the corresponding PubMed ID, title, abstract, EBI link, and publication year were retrieved and incorporated into the text. The text of the abstracts referenced by 16 or more studies was excluded from the analysis to avoid including non-study-specific texts and to prevent skewing document-relatedness comparisons (see “Supplementary Material: 5. Excluded abstract text”). 

MGnify profile data is separated into Taxonomic and Functional files (taxonomy abundances file and GO abundances files, respectively). Taxonomic files are separated into “taxonomy_abundances_LSU” and “taxonomy_abundances_SSU”. The Large Subunit (LSU) comprises 23S sequences in prokaryotes and 28S sequences in eukaryotes, while the Small Subunit (SSU) corresponds to 16S in prokaryotes and 18S in eukaryotes. The file, which comes in tsv format, includes the taxonomy in the first column, the various Runs or Assemblies per sample in the first row, and the count in each cell. Functional files are either the simplified “GO-slim_abundances” (suited for metagenomic data [36]) or the full “GO_abundances” files [6]. This tsv-formatted file includes the columns GO-term, description, and category (molecular function, biological process, or cellular component) in the first three columns, the Run/Assembly IDs in the subsequent columns, and the respective count in each cell. GO terms were selected as they provide a structured, hierarchical vocabulary that integrates functional information across the diverse samples. Pathway-based annotations such as KEGG, eggNO, and KOfam emphasize curated metabolic pathways and ortholog groups. Nonetheless, GO supports broader biological processes, molecular functions, and cellular components characterization, leading it to be widely used in metagenomics. Moreover, MGnify introduces functional annotation steps using InterProScan to assign proteins functions, after which gene ontology (GO) terms are assigned via in-house scripts [36], ensuring thus compatibility and enabling cross-study comparisons and scalable embedding of functional information. All profile data, whether taxonomic or functional, were stored using a metadata-integrated format to enable downstream metadata-matching and similarity calculations.

### Encoding Strategies for Taxonomic and Functional Profiles

**Fig. 7:**
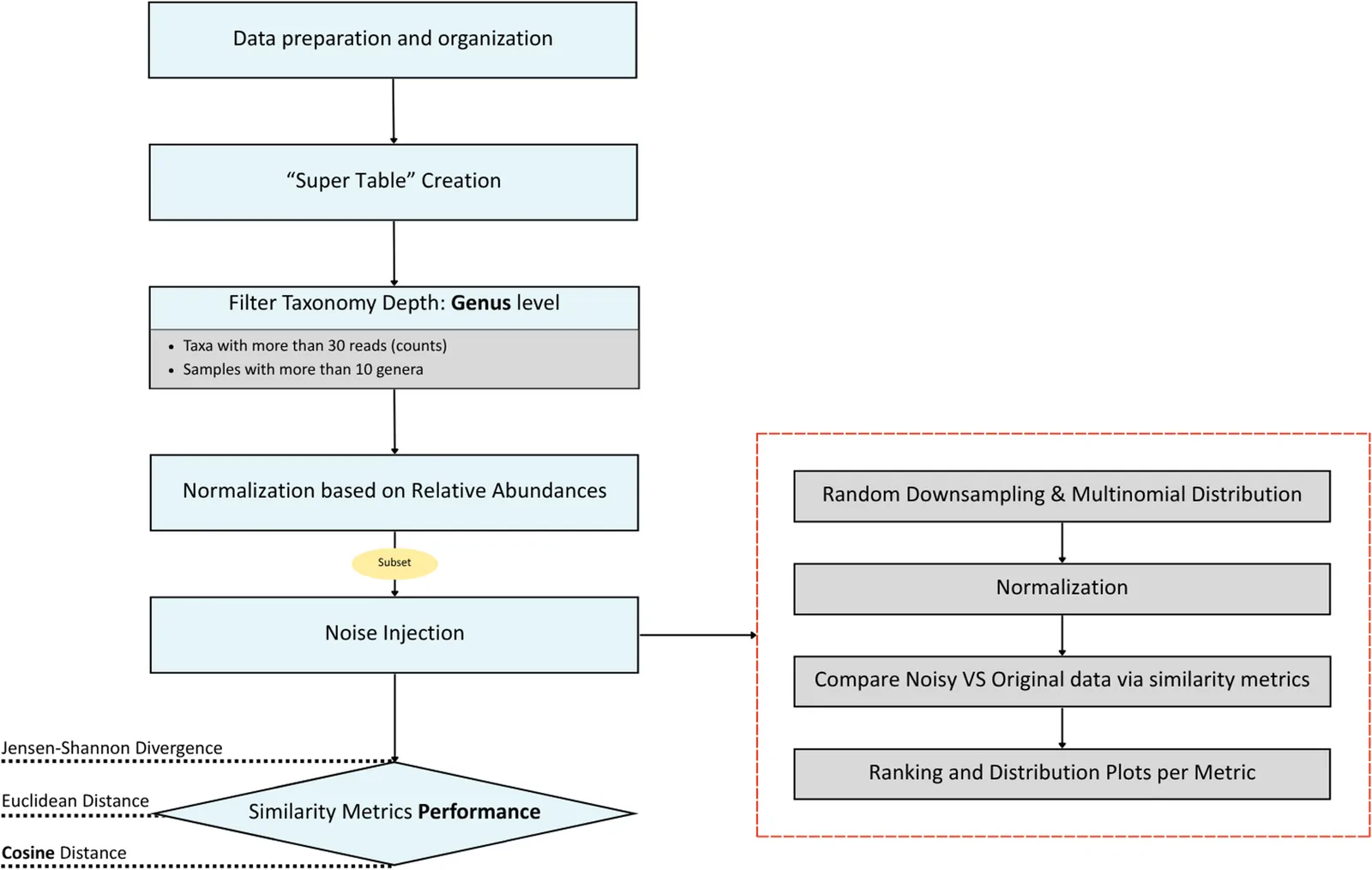
Taxonomic Profiles - Sample Level Workflow. Initially, data is retrieved and organized into abundance tables per MGnify version, which are filtered by taxonomic depth (at the genus level) and normalized based on relative abundances. Similarity metrics, such as Euclidean distance, Jensen–Shannon divergence, and cosine similarity, are used to compare samples globally. These metrics are assessed for their robustness (diamond) against noise-injected data based on downsampling. The effectiveness of these metrics is assessed by a ranking scoring system (right insert)

**Fig. 8:**
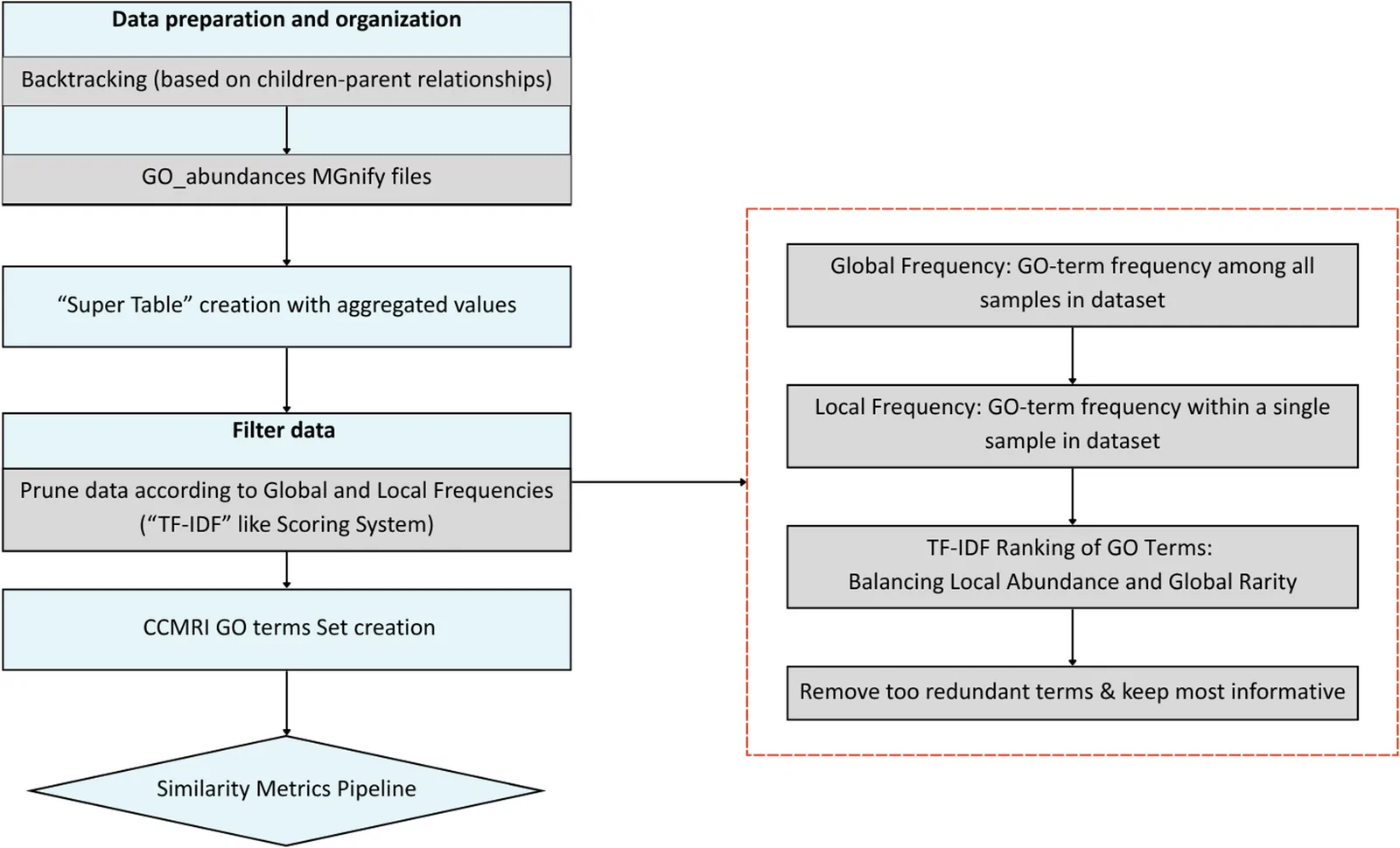
Functional Profiles - Sample Level Workflow. The pipeline begins with data preparation, including backtracking and collection of Gene Ontology (GO) term abundances from MGnify outputs. A “super table” of aggregated functional data is constructed, followed by a filtering stage based on both global (across-sample) and local (within-sample) frequencies of GO terms. A TF-IDF-inspired scoring system is applied to balance term specificity and informativeness, prioritizing GO terms that are abundant in individual samples but rare across the dataset. These scores inform a hierarchical pruning process that eliminates redundant GO terms based on GO structure, resulting in the CCMRI GO Terms Set. This curated, non-redundant set is then used for downstream similarity metrics analysis

To analyze taxonomic and functional profiles across various studies, two structured multi-step workflows were developed and implemented, which are analytically described below and illustrated in Fig. 7 and 8, respectively.

For** taxonomic **data the initial stage involved preprocessing and organizing the retrieved data. To this end, a comprehensive table referred to as the “super table” was constructed for the MGnify 4.1 and 5.0 versions. This table is formatted in long format, where each row consists of a taxon name, a corresponding Run or Assembly ID, and its associated count, enabling thus centralized consolidation of abundance data across all samples for a given version. 

Simultaneously, to ensure **functional** profiles reflect hierarchical relationships among Gene Ontology (GO) terms, a custom backtracking approach was applied. This method aggregates raw data from GO-abundances files by propagating counts from more specific child terms up to their broader parent terms based on the GO hierarchy. By recursively assigning child term abundances to all relevant parent terms, the resulting dataset captures biologically meaningful comparisons across samples, aligning with the structured nature of the GO classification. After completing this step, a “super table” was constructed for each MGnify version (v4.1 and v5.0) to consolidate functional abundance data. This table is organized in long format, where the first column represents the GO term, the second column the corresponding Run/Assembly ID, and the third column the associated count. This structure enables centralized aggregation of GO term abundances across all samples within a given version, facilitating further downstream analysis.

### Preprocessing and Quality Filtering

To ensure the quality of downstream analysis and avoid the influence of low-quality or noisy samples, both data types were subject to extensive filtering. For **taxonomic** data, filtering was applied to control the depth of taxonomy included in the analysis. Given the large volume of data and the potential for incomplete taxonomic hierarchies, it was essential to limit the analysis to a level that balances detail with interpretability. The genus level was selected as it sufficiently captures taxonomic diversity across samples while avoiding too specific or inconsistently labeled taxonomic entries. We additionally applied two filtering criteria to ensure sufficient taxonomic diversity and data reliability for downstream analyses. First, taxa represented by fewer than 30 reads were removed. Low-abundance features in this range are frequently associated with sequencing noise, index bleed, or sampling stochasticity to improve robustness [37]. Second, samples containing fewer than ten genera were excluded, as extremely low richness often reflects technical issues such as insufficient sequencing depth or failed library preparation rather than true biological signal [38]. Together, these thresholds ensure that downstream diversity and similarity analyses are based on stable, informative, and biologically meaningful community profiles. Lastly, an aggregation step was performed, where counts from lower-level taxa were summed into their higher-level parent taxonomic groups to facilitate easier access to the total count information across a taxonomy branch. 

To enhance the interpretability and biological relevance of the **functional** profiles, a filtering step was applied based on two types of frequencies, namely “local” and “global”. Local frequency was defined as the proportion of reads assigned by MGnify (see Data Retrieval) to a GO term within a sample, while global frequency is described as the proportion of samples in which a GO term appeared. GO terms with extremely high global frequency were removed to eliminate overly generic and broadly distributed functions across samples, offering limited value for distinguishing among microbial communities. Conversely, terms with low local frequency, meaning those contributing minimally within individual samples, were filtered out to reduce noise. This dual-threshold filtering ensured that the resulting profiles retained informative, sample-relevant features while minimizing the impact of ubiquitous GO terms. Multiple threshold combinations for global and local frequencies were applied to detect the best set of thresholds, based on the amount and the type of GO terms that were removed. To identify the optimal thresholds, we evaluated all global and local frequency combinations in a comparative table recording (i) the number of GO terms removed for being overly generic/common (high frequency), (ii) the number of GO terms removed for being overly specific or uninformative (low frequency), and (iii) the remaining unique GO terms and run/assembly IDs. This assessment allowed us to balance the removal of generic and specific terms, while avoiding excessive information loss. The selected thresholds (0.75 for global frequency and 0.01 for local frequency) were those that best preserved a stable and biologically meaningful set of GO terms for downstream analysis. A Term Frequency - Inverse Document Frequency (TF-IDF)-based scoring approach was applied to further refine the set of GO terms. The Term Frequency (TF) was calculated as the local frequency explained beforehand, while the Inverse Document Frequency (IDF) was computed as the logarithm of the total number of samples divided by the number of samples in which each GO term appeared. This approach prioritized terms that were both abundant within individual samples and relatively rare across the entire dataset. The resulting TF-IDF score, obtained by multiplying TF by IDF, served as a ranking metric to identify informative and sample-specific GO terms. To reduce semantic redundancy and preserve hierarchical structure, GO terms were further pruned using a top-down approach guided by the Gene Ontology hierarchy. After ranking all GO terms based on their TF-IDF scores in descending order, the highest-scoring term was retained. Subsequently, the algorithm iteratively traversed the ranked list, and for each retained term, all of its descendants (i.e., more specific) terms were removed from further consideration. This hierarchical pruning ensured that no semantically nested or redundant terms remained in the final set. The resulting curated and non-redundant collection of GO terms, referred to as the “**CCMRI Set-of-GO-terms**”, represents a minimal yet highly informative subset optimized for comparative and downstream functional analyses.

In both functional and taxonomic datasets, after applying filtering and removing the relative terms, the final step - prior to similarity metrics calculation - involved normalization of the dataset based on relative abundances. Relative abundance was calculated by dividing the count of each taxon or GO-term within a sample (Run/Assembly ID) by the total taxon/GO-term count for that sample. This normalization ensured that the sum of relative abundances for each sample equaled one, thereby allowing for meaningful comparisons across samples with varying sequencing depths.

### Robustness Evaluation with Controlled Noise Injection

To simulate varying levels of technical and biological variability in count-based data, we introduced controlled noise by emulating reduced sequencing depth while preserving the relative abundance profiles within each Run/Assembly ID. This was achieved using a multinomial distribution, which probabilistically redistributes counts across features according to their original relative proportions, introducing realistic stochastic variation. A subset of 1,000 studies was randomly selected as a test set. For the robustness evaluation step, a subset of 4,000 samples (Run/Assembly IDs) was used for the taxonomic dataset and 2,000 samples (Run/Assembly IDs) for the functional dataset. We then iterated over a series of predefined downsampling ratios (i.e., 0.1, 0.25, 0.75, and 0.9), where each ratio indicates the proportion of Run/Assembly IDs that were affected by the noise injection. For instance, a ratio of 0.25 implies that 25% of the IDs are downsampled, and for each of these IDs the total number of retained counts is reduced to 25% of the original sequencing depth, while the remaining 75% retain their original counts. Consequently, lower ratios correspond to minor alterations across the dataset, whereas higher ratios reflect more extensive noise. This down-sampling strategy was applied, as a common approach in microbiome studies to mitigate biases associated with uneven read counts [39].

**Fig. 9:**
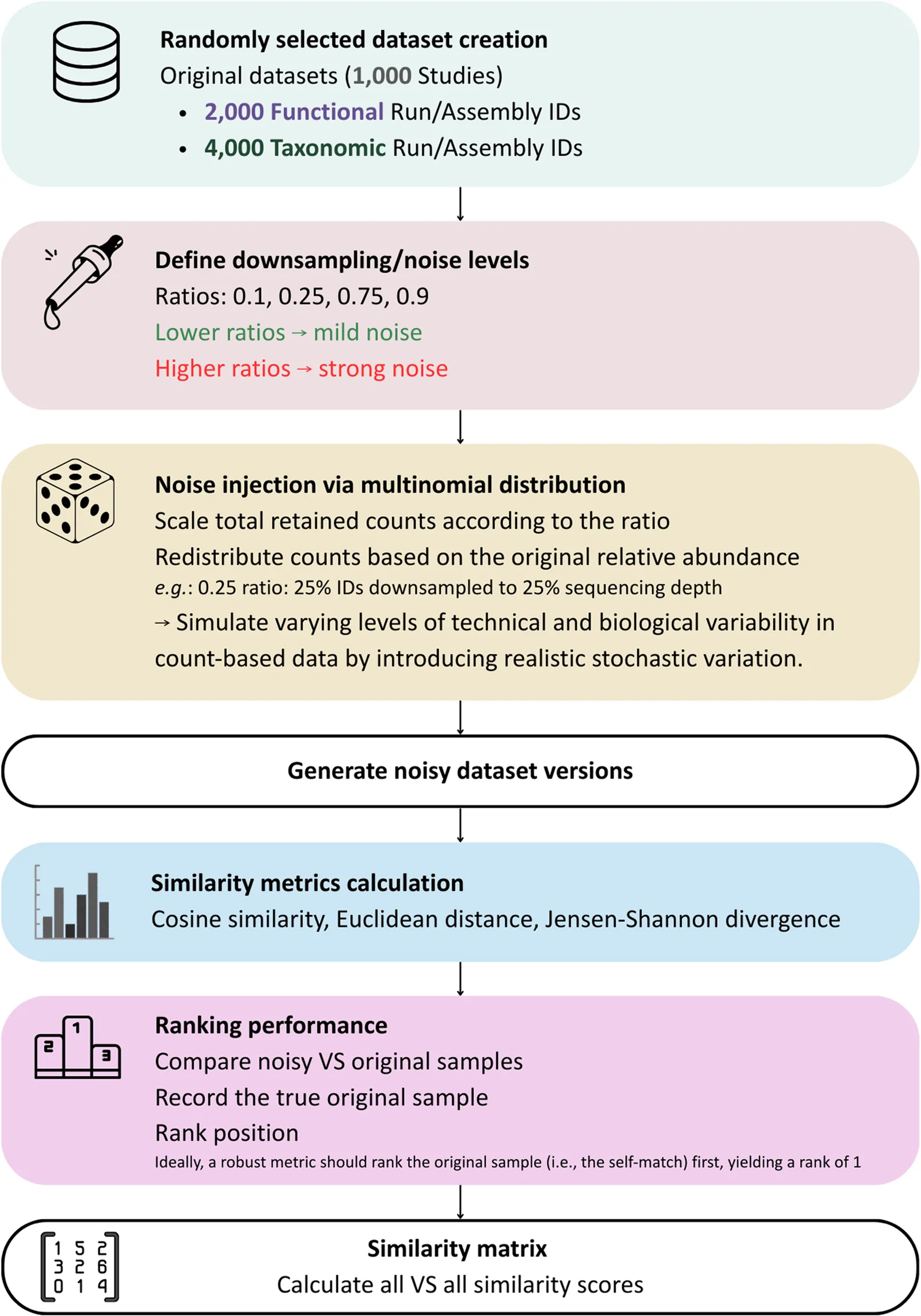
Flowchart describing noise injection via multinomial distribution across predefined downsampling ratios and subsequent evaluation of similarity metrics using ranking-based performance to identify the most robust measure (cosine similarity) for final all-vs-all comparisons

For each affected Run/Assembly ID, counts were resampled using a multinomial distribution, where the total number of retained counts was scaled according to the ratio, and counts were redistributed based on their original relative abundance. After noise injection, we evaluated the similarity between the original and noisy profiles using three metrics, chosen based on their complementary properties and suitability for large-scale data: Euclidean distance as the simplest and oldest traditional metrics, cosine similarity, and Jensen–Shannon divergence, as they are reported for their scalability [16]. Other metrics, such as Bray–Curtis and Jaccard, were also considered but not included for several reasons. Bray–Curtis is known to be unstable under noise and sparsity, particularly when the biological signal is weak relative to sampling depth [40]. In addition, Jaccard is a presence–absence metric and therefore discards quantitative abundance information that is critical for metagenomic analyses [41]. While presence–absence metrics can be more robust to certain types of noise, we specifically chose abundance-based metrics to take full advantage of the count data provided by MGnify, capturing the richness and quantitative structure of microbial communities. The objective was to assess the ability of each metric to correctly match noisy samples with their original counterparts. We implemented a ranking-based evaluation, where each noisy sample was compared against all original samples, and the rank position of the true original was recorded. Ideally, a robust metric should rank the original sample (i.e., the self-match) first, yielding a rank of 1. We extended this evaluation to top-k ranking scenarios (e.g., top-1, top-3, top-5), quantifying the number of cases where the correct match appeared within the top-k most similar profiles. Top-k ranking approach is considered to be a classical ranking algorithm for various information retrieval, machine learning, and similarity benchmarking tasks [42]. This framework enabled a comparative analysis of metric performance, allowing us to identify the most reliable similarity measure for capturing signal preservation under noise. After selecting the optimal metric, pairwise cosine similarities were calculated between all Run/Assembly IDs within each version, employing an “all vs. all” comparison approach. The flowchart of the noise injection pipeline is presented in Fig. 9.

The statistics of the final taxonomic data are represented in Table 1, while for functional data are shown in Table 2. 

**Table 1:** Summary of Final Taxonomic Dataset and Pairwise Comparison Counts. This table presents the final dataset composition following taxonomic filtering, organized by MGnify version (v4.1 and v5.0) and rRNA marker type (LSU and SSU). For each dataset version, the table lists: (i) the number of studies processed, (ii) the number of unique samples retained after filtering, and (iii) the total number of all-vs-all pairwise comparisons, calculated using the formula *n(n–1)/2*, where n is the number of samples. This comprehensive comparison matrix forms the basis for subsequent similarity calculations. The selected versions represent the most recent and high-quality releases available for each marker type

Version	Studies	Unique filtered samples	Number of comparisons [n * (*n*−1)/2]
v4.1_LSU	1,395	8,151	33,215,325
v4.1_SSU	2,302	157,906	12,467,073,465
v5.0_LSU	663	1,037	537,166
v5.0_SSU	972	58,319	1,700,523,721

**Table 2:** Summary of Final Functional Dataset and Pairwise Comparison Counts. This table summarizes the final dataset used for functional analyses, based on MGnify versions v4.1 and v5.0. For each version, the number of studies included, the total number of unique samples retained after functional filtering, and the resulting number of all-vs-all pairwise comparisons are reported. Pairwise comparisons were computed using the formula *n(n–1)/2*, where n is the number of filtered samples. These comparisons provide the foundation for calculating functional similarities and conducting clustering analyses across microbial communities

Version	Studies	Unique filtered samples	Number of comparisons [n * (*n*−1)/2]
v4.1	1,063	64,503	2,080,286,253
v5.0	480	31,962	510,768,741

### Sample-to-Study Aggregation of Similarities

To enable higher-level comparisons between studies, individual sample-level similarity matrices were aggregated into study-level similarity matrices. Each study, composed of multiple samples, was represented by summarizing its pairwise similarities with all samples from another study in both directions. This approach condenses the n × n similarity matrix (where n is the number of samples) into a smaller m × m matrix (m being the number of studies), allowing for clearer cross-study comparisons. We used a nearest-neighbor-based approach, in order to emphasize the closest cross-study relationships. 

In practice, the similarity between two studies was calculated by first computing the pairwise similarities between all samples in study X and all samples in study Y. For each sample in study X, the maximum similarity to all samples in study Y was calculated. These maximum similarities were then averaged yielding an aggregated similarity X → Y. The same procedure was performed in the opposite direction to obtain the aggregated similarity Y → X. Formally:

- X → Y:
  - For each $x\;\varepsilon\;X$ and $y\;\varepsilon\;Y$ :$\left(x,\;Y\right)$ = max $\sim\left(x,y\right)$
- Y → X:
  - Analogously, for each $x\;\varepsilon\;X$ and $y\;\varepsilon\;Y$:$\left(y,\;X\right)=$ max $\sim\left(y,\;x\right)$ 
- Study-level
  - $\left(X,\;Y\right)$ =max (mean($\sim\left(x,y\right))$ , mean (*∼(y,X)))*

Below in Table 3 is presented an example of this approach.

**Table 3:**
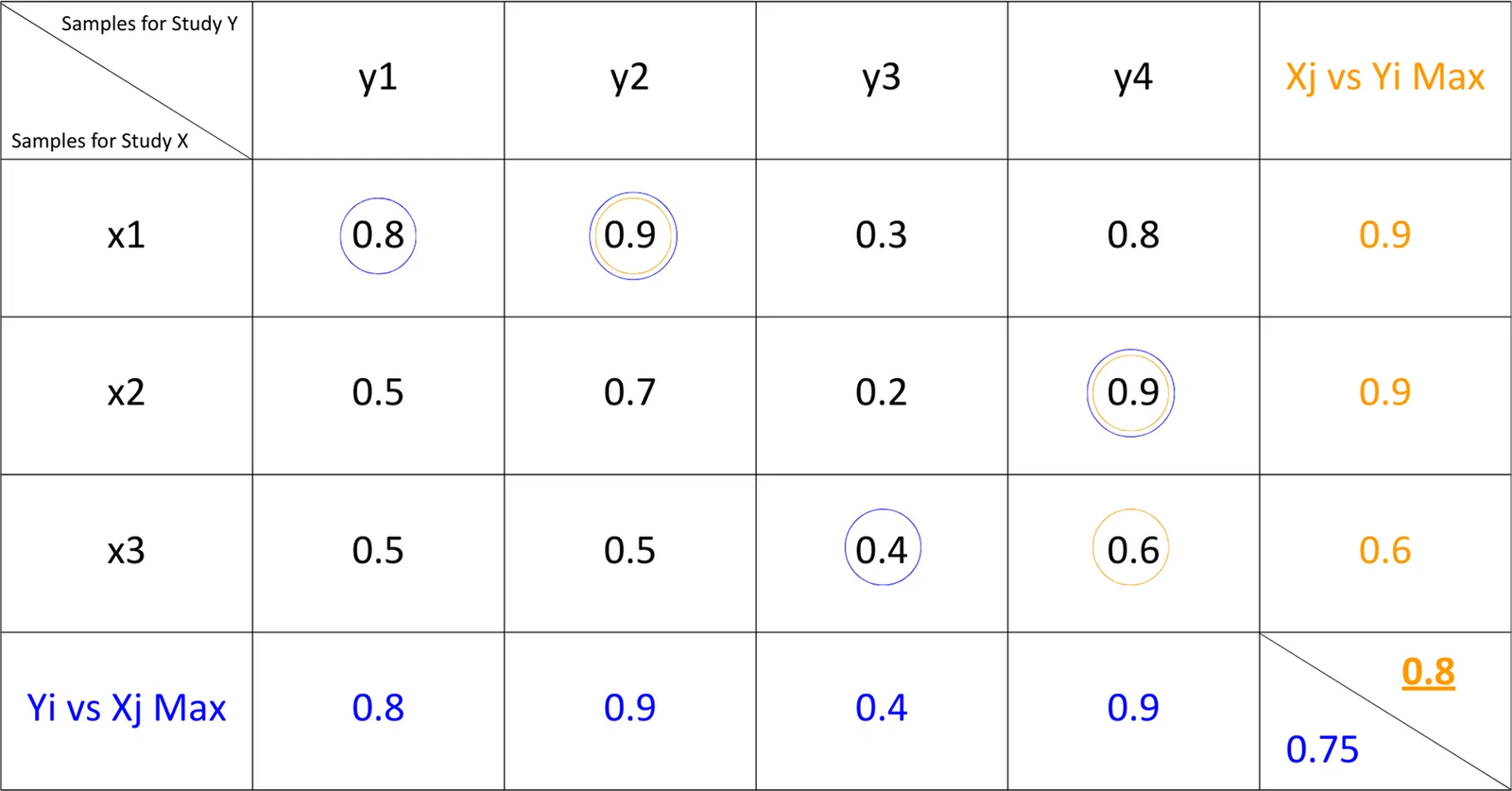
Example of aggregating sample-level similarities to compute the study-level similarity matrix. Pairwise similarities between samples from Study X (x₁–x₃) and Study Y (y₁–y₄) are shown. For each sample in Study X, the highest similarity to any sample in Study Y is identified, and these per-sample maxima are averaged to yield the directional similarity X → Y. The same procedure is applied in the opposite direction to obtain Y → X. The final study-level similarity (bottom right) is defined as the maximum of these two directional averages, emphasizing the strongest cross-study concordance

This nearest-neighbor-averaged, direction-aware procedure provides a comprehensive summary that prioritizes the strongest biological concordance between studies. The approach ensures that shared biological structure (e.g. common controls, shared environments, similar functional profiles) is retained even when other sample groups differ substantially. In this way, the method reduces information loss by preserving the best-matching relationships between studies rather than diluting them across all pairwise comparisons. At the same time, if only part of the two studies overlaps (e.g. similar controls but different treatments), the directional averages will differ, and the final score will reflect only partial similarity rather than collapsing the studies into an artificially high or low value.

The resulting matrix is symmetric, and each cell reflects the maximum functional or taxonomic similarity between two studies, offering a more robust and interpretable overview at the study level while preserving the underlying sample-level variation. 

### Semantic Relatedness via Large Language Models

To further assess the semantic relatedness between metagenomic studies based on their text, we leverage LLMs. Specifically, for each microbiome study, textual information, including title, description, and biome, along with the linked PubMed abstract, was retrieved from the MGnify database. This structured metadata allows comparison of ecological contexts across studies at varying levels of resolution (e.g., aquatic vs. freshwater vs. sediment). This approach enables us to facilitate whole systems assessment by providing an efficient first-pass filter for curating similar/dissimilar studies. 

LLM-based study similarity was performed using Ollama v0.6.8 deployed on the LifeWatch ERIC HPC AI server. A custom pipeline was implemented in bash for process orchestration and Python v3.5 for model invocation and study comparison routines. All MGnify study IDs and their associated textual metadata/literature were provided as input. The codebase was optimized to take full advantage of the available GPU resources on the HPC system by running parallel processes and multiple concurrent Ollama server instances. This configuration enabled scalable and efficient inference across large numbers of pairwise study comparisons.

For our task, we adopted a prompt engineering approach, which required the use of an instruction-tuned model capable of understanding and responding to natural language instructions. While models like SciBERT and other BERT-based models were pre-trained with masked-language modeling and excel at generating domain-specific embeddings ([43, 44]), they are not inherently designed for prompt-driven reasoning tasks without extensive fine-tuning ([44]). We evaluated two computationally efficient, instruction-tuned and GPU-compatible LLMs available through the Ollama platform: Qwen-3 30B-A3B (qwen3:30b-a3b-q8_0) and Phi-4 14B (phi4:14b-q8_0). Qwen-3 is a substantially larger Mixture-of-Experts model with ~ 30.5B total parameters (≈ 3.3B active per token) and support for very long contexts of up to ~ 131 K tokens. In contrast, Phi-4 is a 14B-parameter dense model with a 16 K-token context window, trained on a carefully curated dataset with strong reasoning-oriented fine-tuning.

Despite Qwen-3’s higher capacity, larger parameter budget, deeper architecture, and broader multilingual training corpus, Phi-4 consistently outperformed it on our task. The latter provided stronger reasoning quality, more accurate interpretation of prompt intent, more reliable instruction-following, and more consistent, structured, logic-driven explanations of semantic similarity between input texts. These qualities were essential for downstream automation and reproducibility, leading us to select Phi-4 as the primary model.

Phi-4 (14B parameters, quantization level Q8_0, pretrained as of February 2025 [45]) was deployed and managed via the Ollama platform. The prompt shown below was developed in a stepwise fashion through iterative qualitative evaluation by inspecting the LLMs’ responses to the same study pairs. Evaluation focused on response extent, contextual grounding, and decisiveness, rather than exhaustive prompt optimization. Earlier prompts tended to produce more ambiguous classifications (e.g., “likely”, “unlikely”), whereas the final prompt yielded more confident, analytical and categorical judgments (e.g., labels of relatedness “None”, “Low“, “Medium”, “High”). This shift appears to be driven by the inclusion of explicit example criteria for each category, which provided clearer guidance on how distinctions should be made. As a result, the final prompt consistently generated more structured, narrative-style explanations with a scientific tone and clearer justification of similarities and differences between study pairs, increasing confidence in outputs. 

The core question presented to the LLM, along with detailed instructions on how to assess relatedness and categorize it into four levels (high, medium, low, and no), is shown below:

How high similarity do you expect between these two microbiome studies? Instructions: Focus on the biomes from which the samples were collected. In the next line quantify the similarity strictly according to these categories: '***high***' (studies of very similar biomes from the same organisms, closely related environments, and similar conditions), '***medium***' (studies of similar biomes from the same organisms or similar environments), '***low***' (studies of the same type of biome but from different organisms or environments) or '***no***' (completely unrelated studies, such as a host microbiome and a soil microbiome).

The full prompt is provided in the “Supplementary Material: 6. LLM full prompt” section. The model’s response, formatted as JSON, includes both an analytical explanation for the assigned relatedness of each study pair and the final relatedness category.

To ensure the robustness and reliability of the model’s assessments, the evaluation was repeated across three separate runs. This approach reduces the inherent stochastic variability of autoregressive LLM outputs. Each study pair was evaluated three times, and a majority-vote strategy was applied to determine the final category. The analysis was performed for 16,194 study pairs for which we had either taxonomic or functional data available.

### Connecting Text-Based Relatedness and Data-Based Study Similarity

A central question is whether and to what extent semantic relatedness inferred from LLMs can reflect biological relatedness based on taxonomic or functional composition. To this end, the similarities and relatedness, as calculated in the previous method sections, had to be combined. This was achieved by plotting the distributions of functional and taxonomic study pair similarities against the different levels of LLM-assessed relatedness (i.e. high, medium, low, none) (see Figures 3–6). For each similarity type and relatedness level, violin plots of similarity values were generated to illustrate how study pairs were distributed along the similarity range (0–1, where a score of 0 indicates no similarity between study pairs, while a score of 1 indicates complete similarity). Separate color palettes were applied for functional and taxonomic comparisons (purple for functional and green for taxonomic) to ensure visual distinction. Python (v3.5) libraries’ pandas, seaborn and matplotlib were used to produce all relative plots. 

### Implementation & Availability

The methodology described was implemented using the Python programming language (v3.5). Multiple packages and libraries were utilized to support data manipulation, statistical analysis, visualization, and Large Language Models. Among the most commonly used were: pandas (v2.2.2) for efficient loading, manipulation, and preprocessing of tabular data; numpy (v2.2.0) for numerical operations and array-based computations; matplotlib (v3.9.2) for generating static visualizations and custom plots; seaborn (v0.13.2) for high-level statistical data visualization and enhancing plots; scipy (v1.14.1) for advanced statistical operations, and lastly scikit-learn (v1.6.0) for machine learning tasks. 

The code is publicly available on GitHub (https://github.com/lab42open-team/similarity_metrics). 

## Supplementary Information

Below is the link to the electronic supplementary material.

Supplementary Material 1 (DOCX 841 KB)
Supplementary Material 2 (DOCX 9.95 KB)

## Data Availability

MGnify data was retrieved via MGnify’s API (as of March 4th, 2024).
