# Supplementary material for: LLM-Assessed Relatedness of Microbiome Study Descriptions Aligns more Strongly with Functional than with Taxonomic Profile Similarity
Source: Microb Ecol. 2026 Mar 31;89(1):104. doi: 10.1007/s00248-026-02730-5 (PMC13171980; doi:10.1007/s00248-026-02730-5)
Supplement: Supplementary file 1 — Supplementary Material 1 (DOCX 841 KB) Supplementary Material 2 (DOCX 9.95 KB) [file 248_2026_2730_MOESM1_ESM.docx]

# Supplementary Material

## 1. Noise Injection

Results for the rest of the top-k ranks (*i.e.,* top-1, top-5, top-10, top-100) for both taxonomic and functional data can be found in this chapter.

First, results for Taxonomic profiles can be found in Figures 10a to 10d.


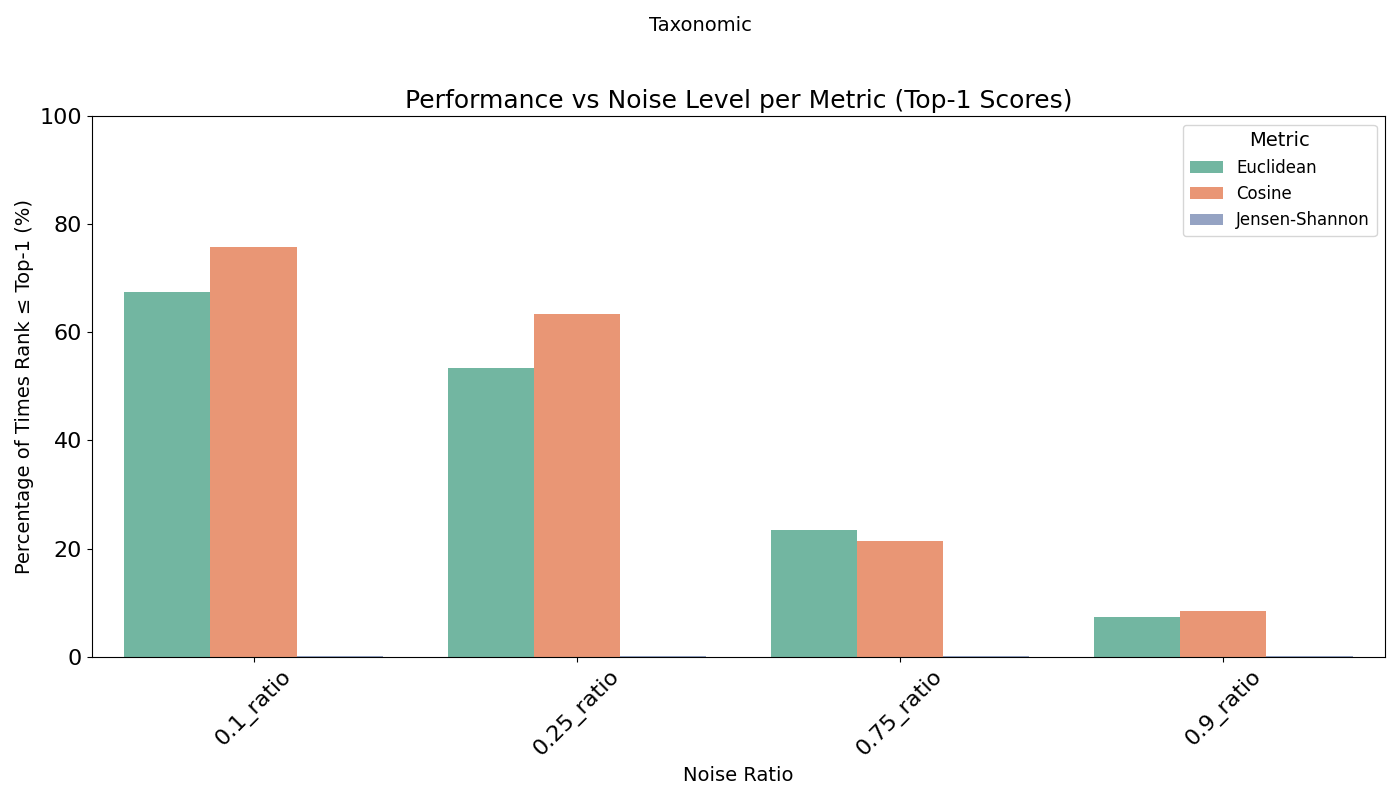


Figure 10a: Top-1 retrieval accuracy under varying noise (0.1, 0.25, 0.75, 0.9) for taxonomic profiles, comparing cosine similarity (orange bar), Euclidean distance (green bar), and Jensen-Shannon divergence (purple bar).


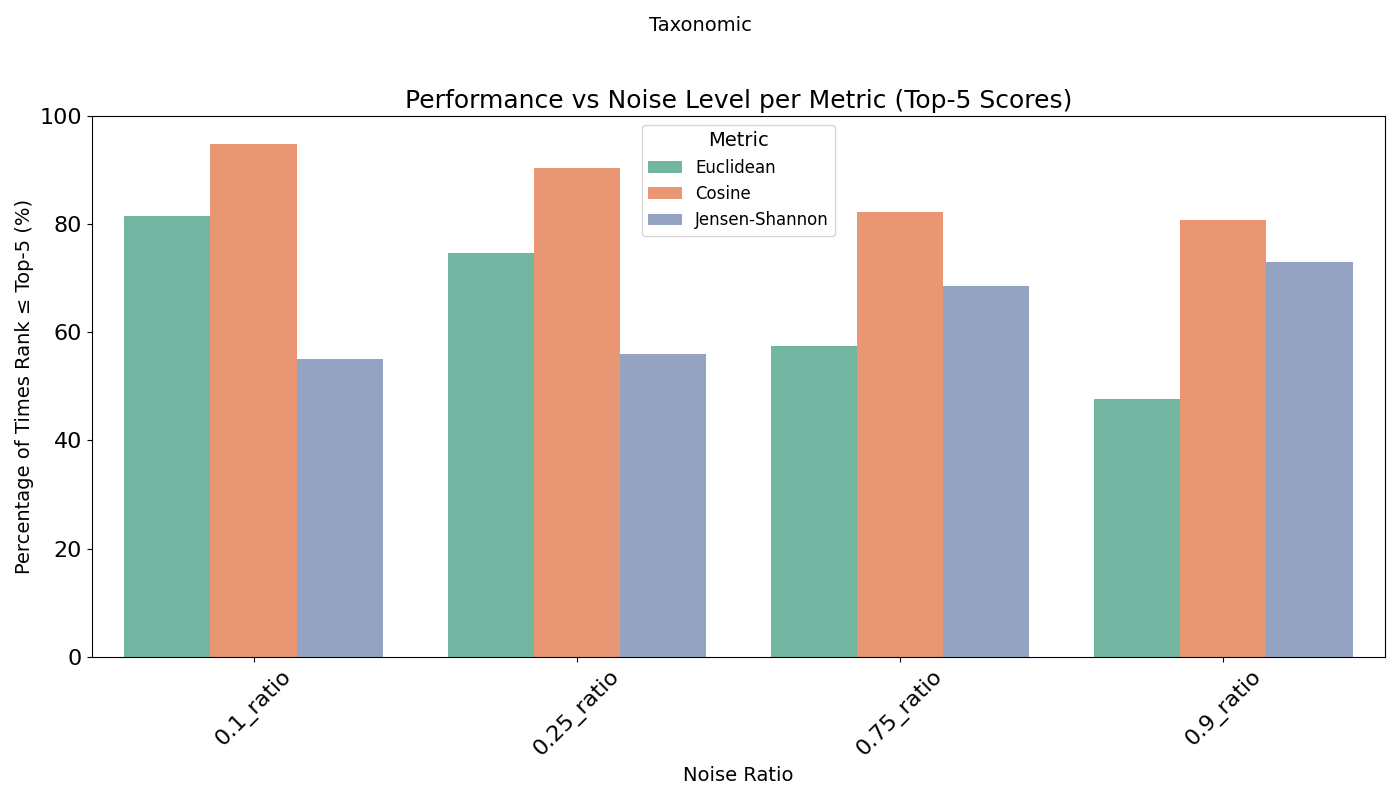


Figure 10b: Top-5 retrieval accuracy under varying noise (0.1, 0.25, 0.75, 0.9) for taxonomic profiles, comparing cosine similarity (orange bar), Euclidean distance (green bar), and Jensen-Shannon divergence (purple bar).


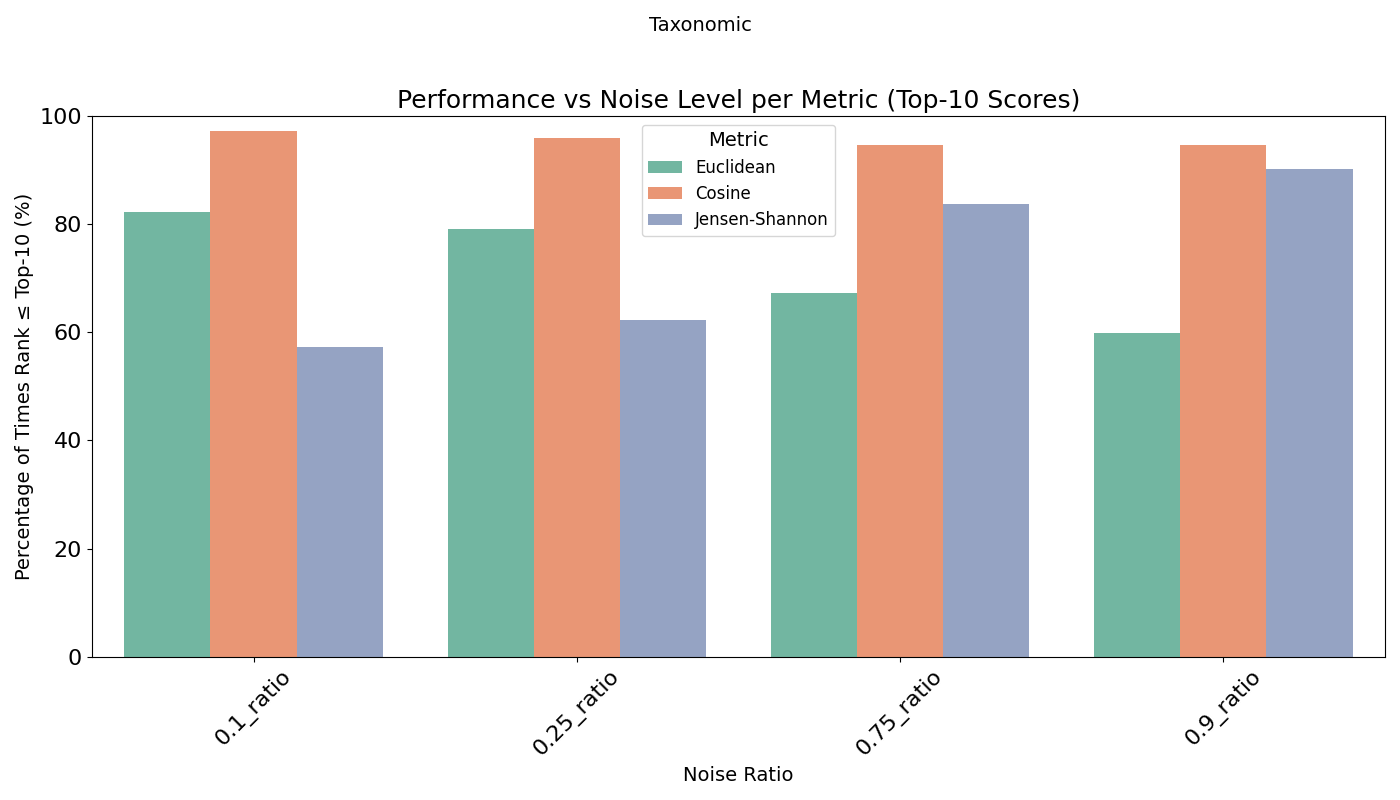


Figure 10c: Top-10 retrieval accuracy under varying noise (0.1, 0.25, 0.75, 0.9) for taxonomic profiles, comparing cosine similarity (orange bar), Euclidean distance (green bar), and Jensen-Shannon divergence (purple bar).


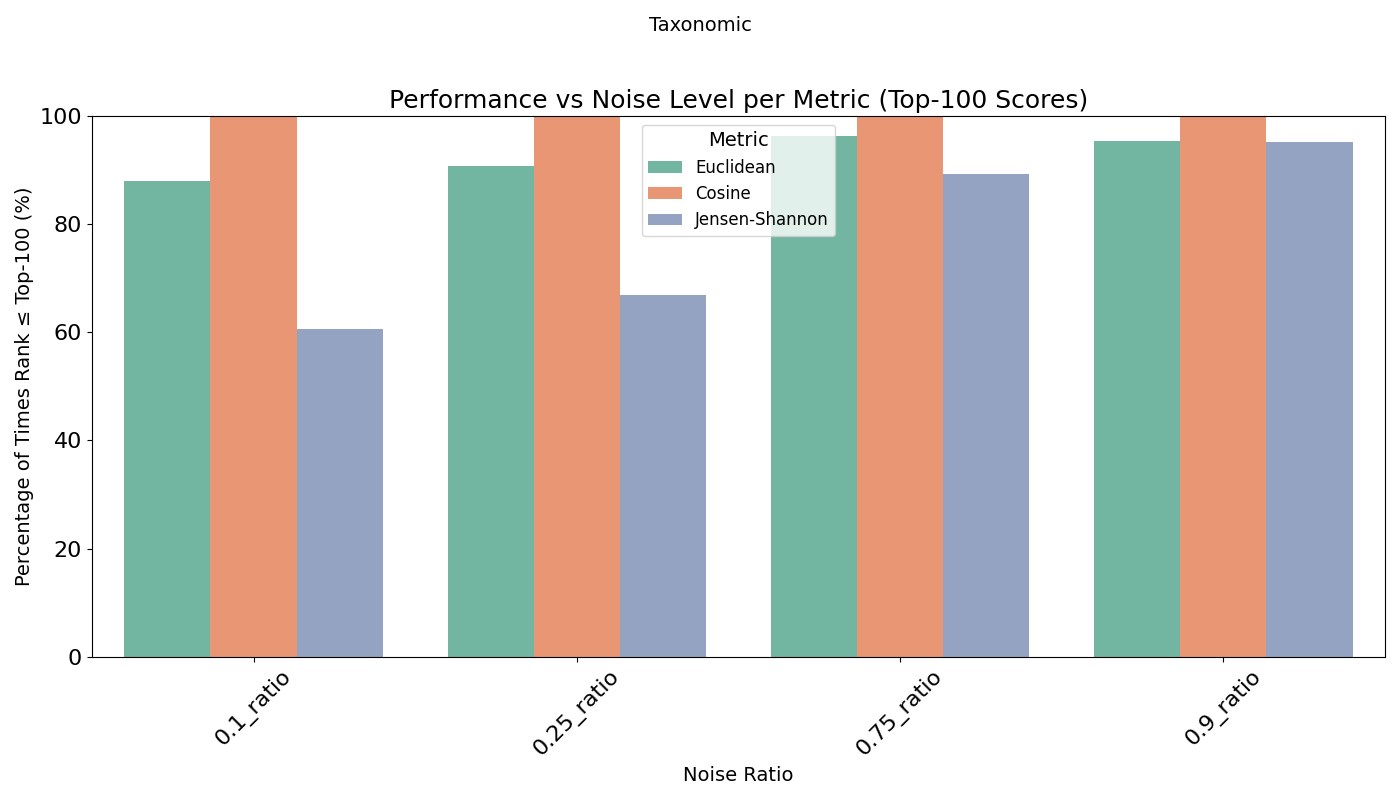


Figure 10d: Top-100 retrieval accuracy under varying noise (0.1, 0.25, 0.75, 0.9) for taxonomic profiles, comparing cosine similarity (orange bar), Euclidean distance (green bar), and Jensen-Shannon divergence (purple bar).

Overall, cosine similarity significantly outperforms both Euclidean distance and Jensen-Shannon divergence on taxonomic data, across different noise levels and top-k ranks.

Similarly, results for Functional profiles follow in Figures 11a to 11d.


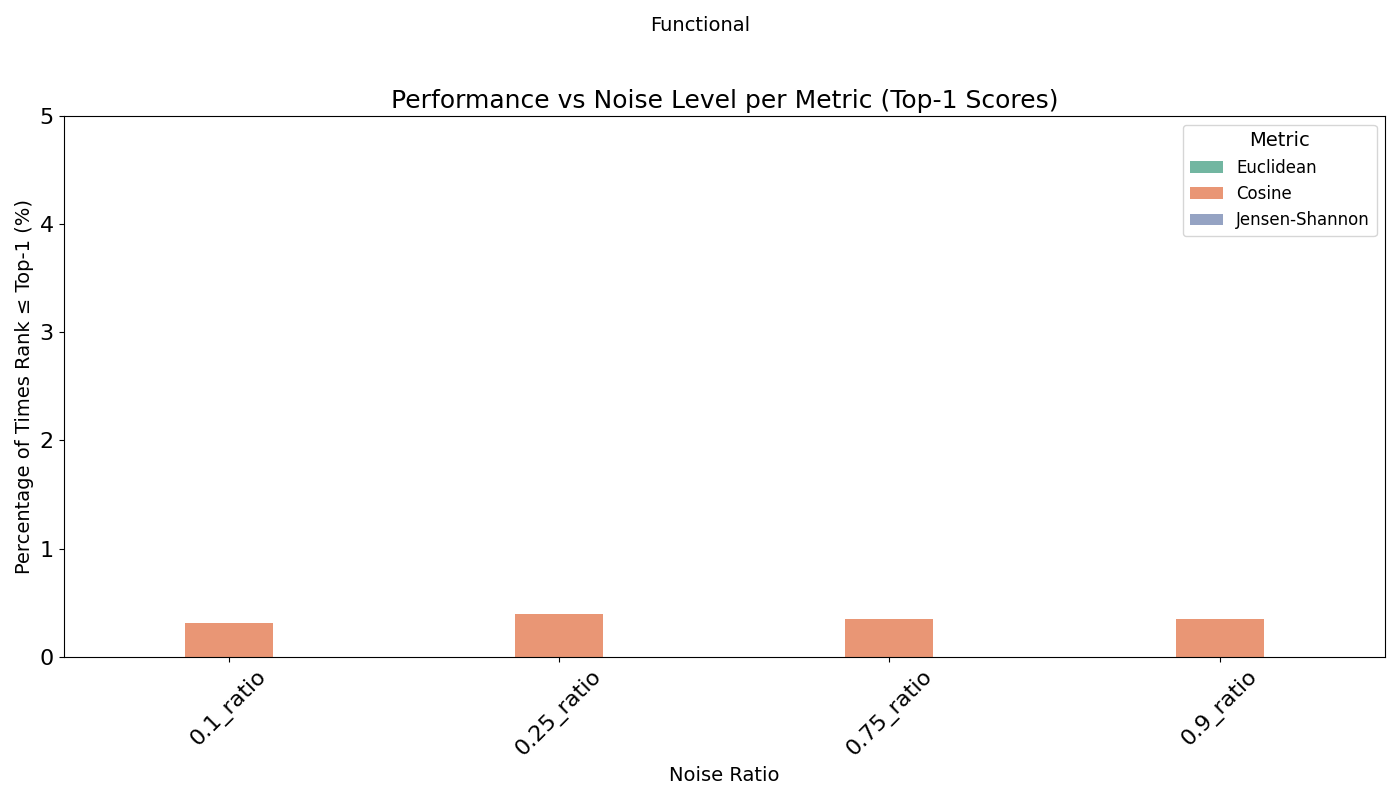


Figure 11a: Top-1 retrieval accuracy under varying noise (0.1, 0.25, 0.75, 0.9) for functional profiles, comparing cosine similarity (orange bar), Euclidean distance (green bar), and Jensen-Shannon divergence (purple bar). The y-axis is limited to 5% to improve visibility of the small accuracy values.


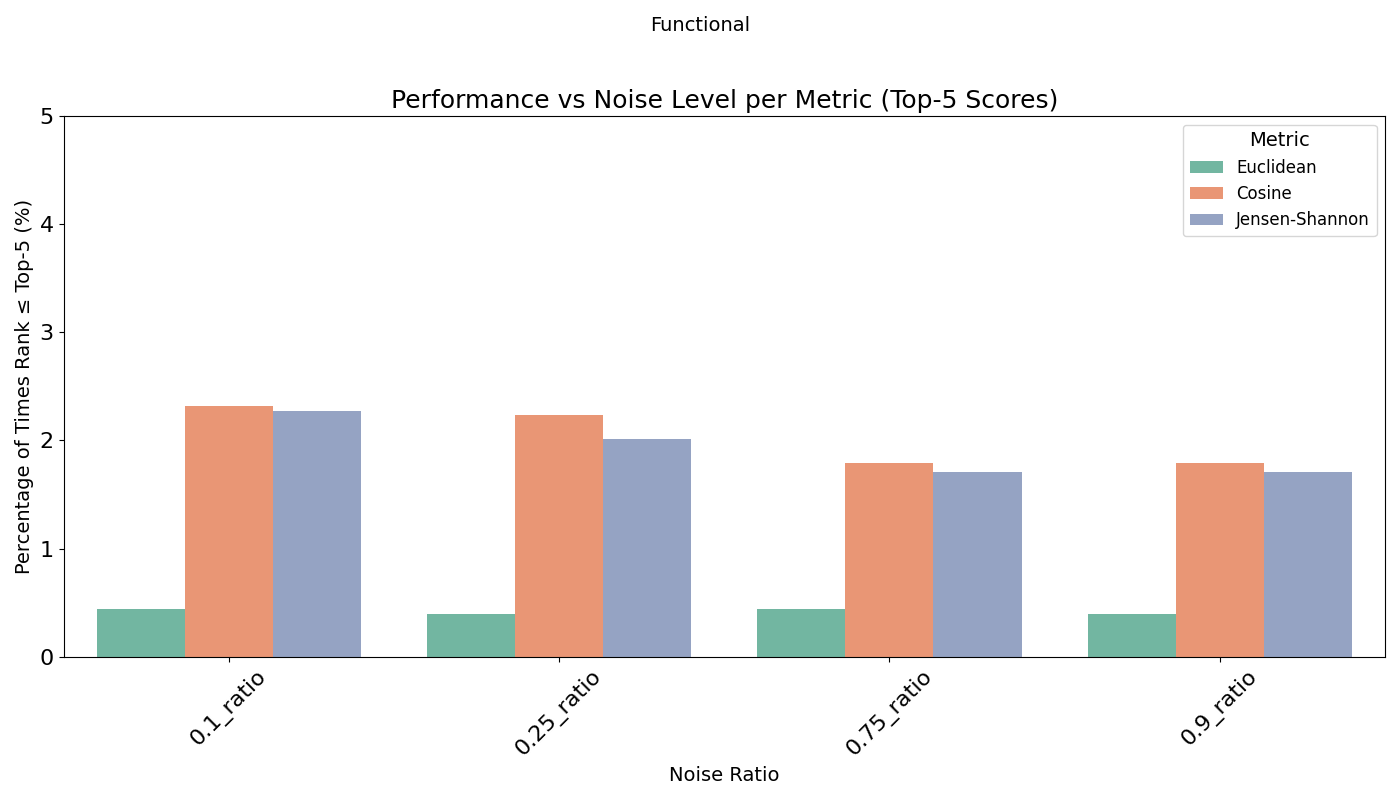
Figure 11b: Top-5 retrieval accuracy under varying noise (0.1, 0.25, 0.75, 0.9) for functional profiles, comparing cosine similarity (orange bar), Euclidean distance (green bar), and Jensen-Shannon divergence (purple bar). The y-axis is limited to 5% to improve visibility of the small accuracy values.


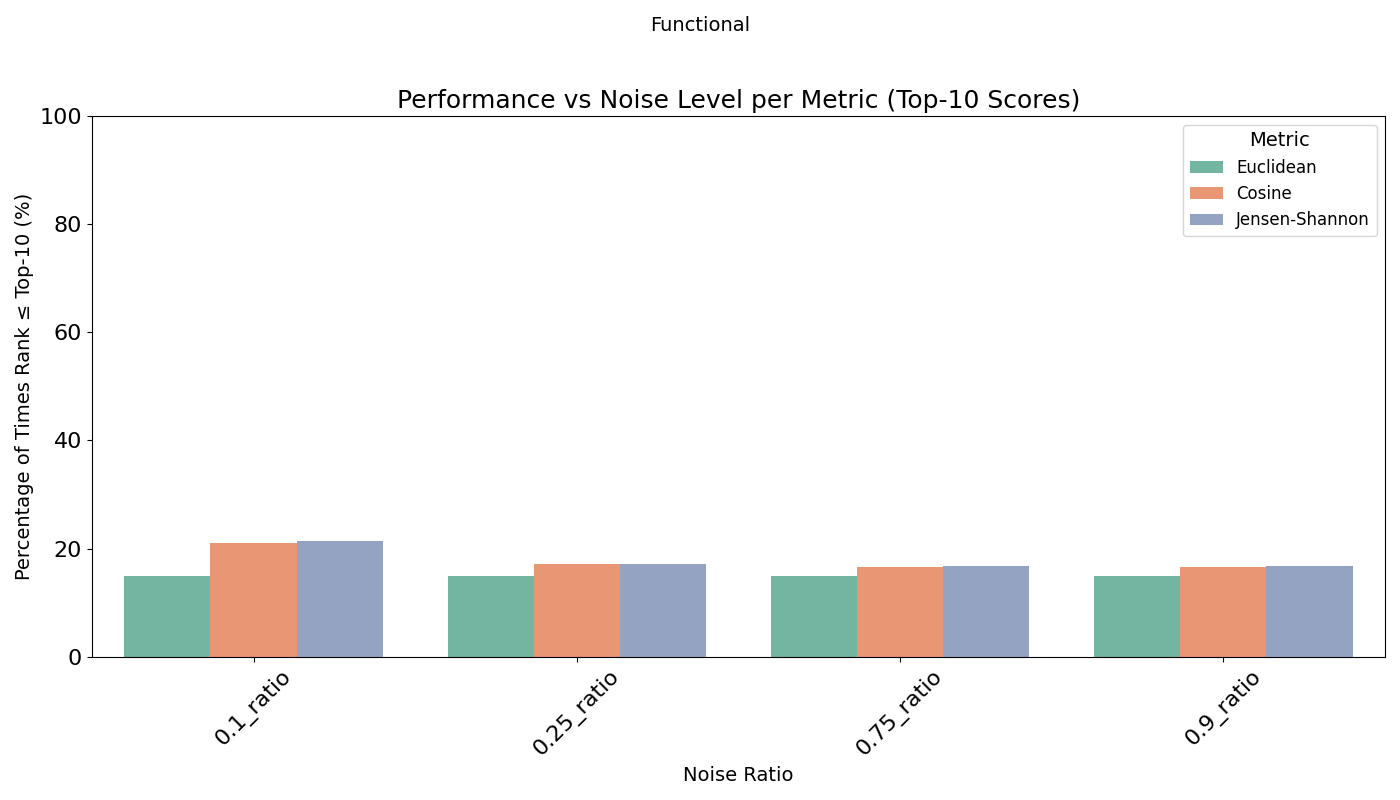


Figure 11c: Top-10 retrieval accuracy under varying noise (0.1, 0.25, 0.75, 0.9) for functional profiles, comparing cosine similarity (orange bar), Euclidean distance (green bar), and Jensen-Shannon divergence (purple bar).


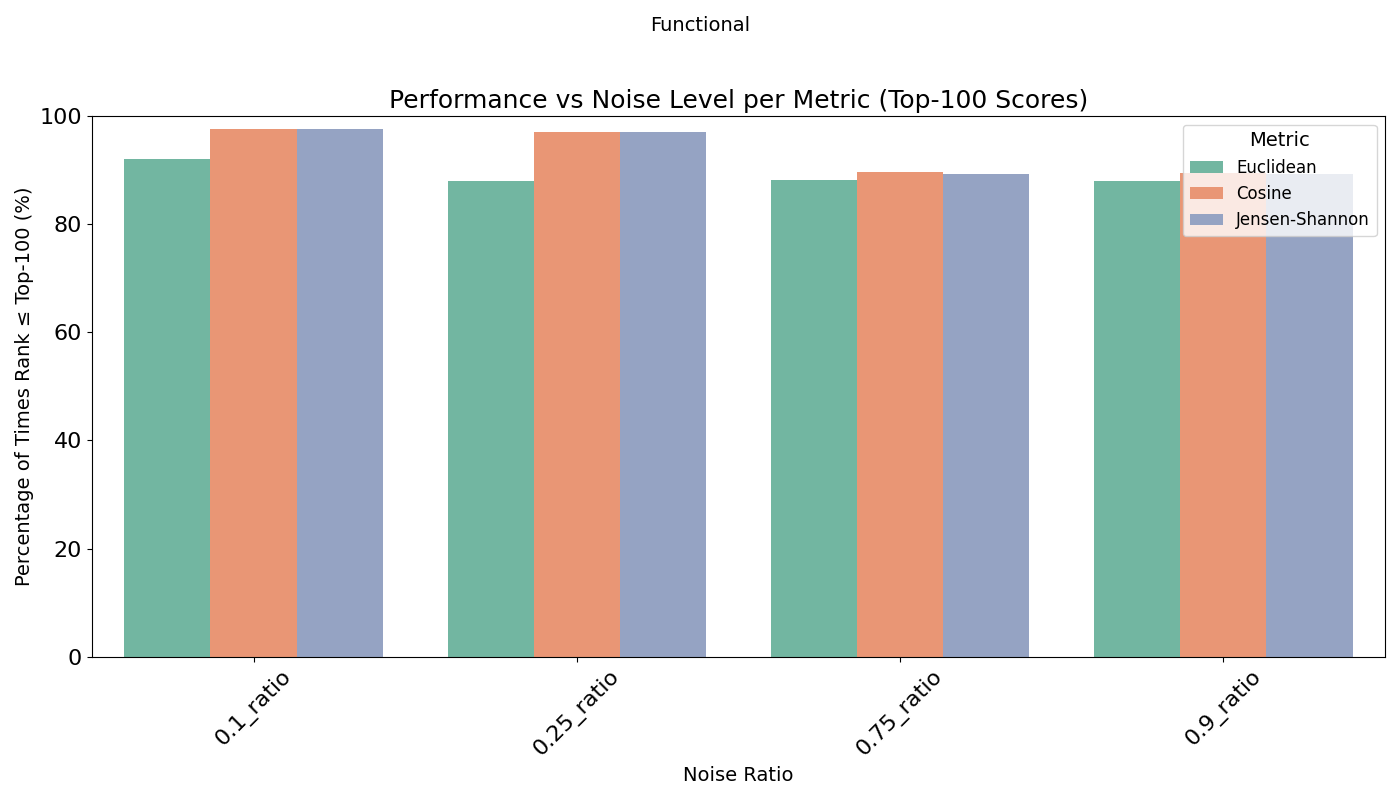


Figure 11d: Top-10 retrieval accuracy under varying noise (0.1, 0.25, 0.75, 0.9) for functional profiles, comparing cosine similarity (orange bar), Euclidean distance (green bar), and Jensen-Shannon divergence (purple bar).

Overall, cosine similarity slightly outperforms both Euclidean distance and Jensen-Shannon divergence on functional data, across different noise levels and top-k ranks.

## 2. Summary of Mann-Whitney U Tests by LLM-Relatedness

| **Similarity**  **Category** | **Comparison** | **n_cat** | **n_rest** | **U_stat** | **p_value_raw** | **p_value_adj** | **reject_H0** |
| --- | --- | --- | --- | --- | --- | --- | --- |
| **run 1** | | | | | | | |
| Taxonomic | high VS medium & low | 853 | 4778 | 2179703 | 0.000945486751828156 | 0.000945486751828156 | True |
|  | medium VS high & low | 2190 | 3441 | 4184300.5 | 9.61559112111077E-13 | 1.23629028699996E-12 | True |
|  | low VS high & medium | 2588 | 3043 | 3379350.5 | 8.05724287023335E-21 | 1.61144857404667E-20 | True |
| Functional | high VS medium & low | 1856 | 1889 | 1216333 | 3.31369130153297E-59 | 1.98821478091978E-58 | True |
|  | medium VS high & low | 961 | 2784 | 1582738.5 | 2.2394664083246E-17 | 3.35919961248691E-17 | True |
|  | low VS high & medium | 928 | 2817 | 1598720.5 | 1.76492225550285E-24 | 4.53837151415018E-24 | True |
| **run 2** | | | | | | | |
| Taxonomic | high VS medium & low | 853 | 4704 | 2171611.5 | 0.0000925512833477998 | 9.79954764859057E-05 | True |
|  | medium VS high & low | 2178 | 3379 | 4074565 | 5.47732062676472E-12 | 6.57278475211766E-12 | True |
|  | low VS high & medium | 2526 | 3031 | 3267963.5 | 8.99931188381959E-22 | 2.02484517385941E-21 | True |
| Functional | high VS medium & low | 2021 | 2070 | 1406923 | 1.67649969106248E-73 | 3.01769944391247E-72 | True |
|  | medium VS high & low | 1053 | 3038 | 1890677 | 1.16717322215416E-18 | 2.10091179987749E-18 | True |
|  | low VS high & medium | 1017 | 3074 | 1956771 | 1.73867687565862E-33 | 7.8240459404638E-33 | True |
| **run 3** | | | | | | | |
| Taxonomic | high VS medium & low | 871 | 4818 | 2302613.5 | 2.95519996013535E-06 | 3.32459995515227E-06 | True |
|  | medium VS high & low | 2140 | 3549 | 4243887.5 | 3.20982575634312E-14 | 4.4443741241674E-14 | True |
|  | low VS high & medium | 2678 | 3011 | 3380897 | 6.78238379473805E-27 | 2.03471513842141E-26 | True |
| Functional | high VS medium & low | 2038 | 2129 | 1480309 | 1.53963750181737E-70 | 1.38567375163563E-69 | True |
|  | medium VS high & low | 1027 | 3140 | 1904290.5 | 2.68387942919482E-18 | 4.3918027023188E-18 | True |
|  | low VS high & medium | 1102 | 3065 | 2086056.5 | 4.08887460814469E-31 | 1.47199485893209E-30 | True |

Table S1: Summary of Mann-Whitney U-tests results per LLM run for taxonomic and functional data.

## 3. LLMs tested per biome


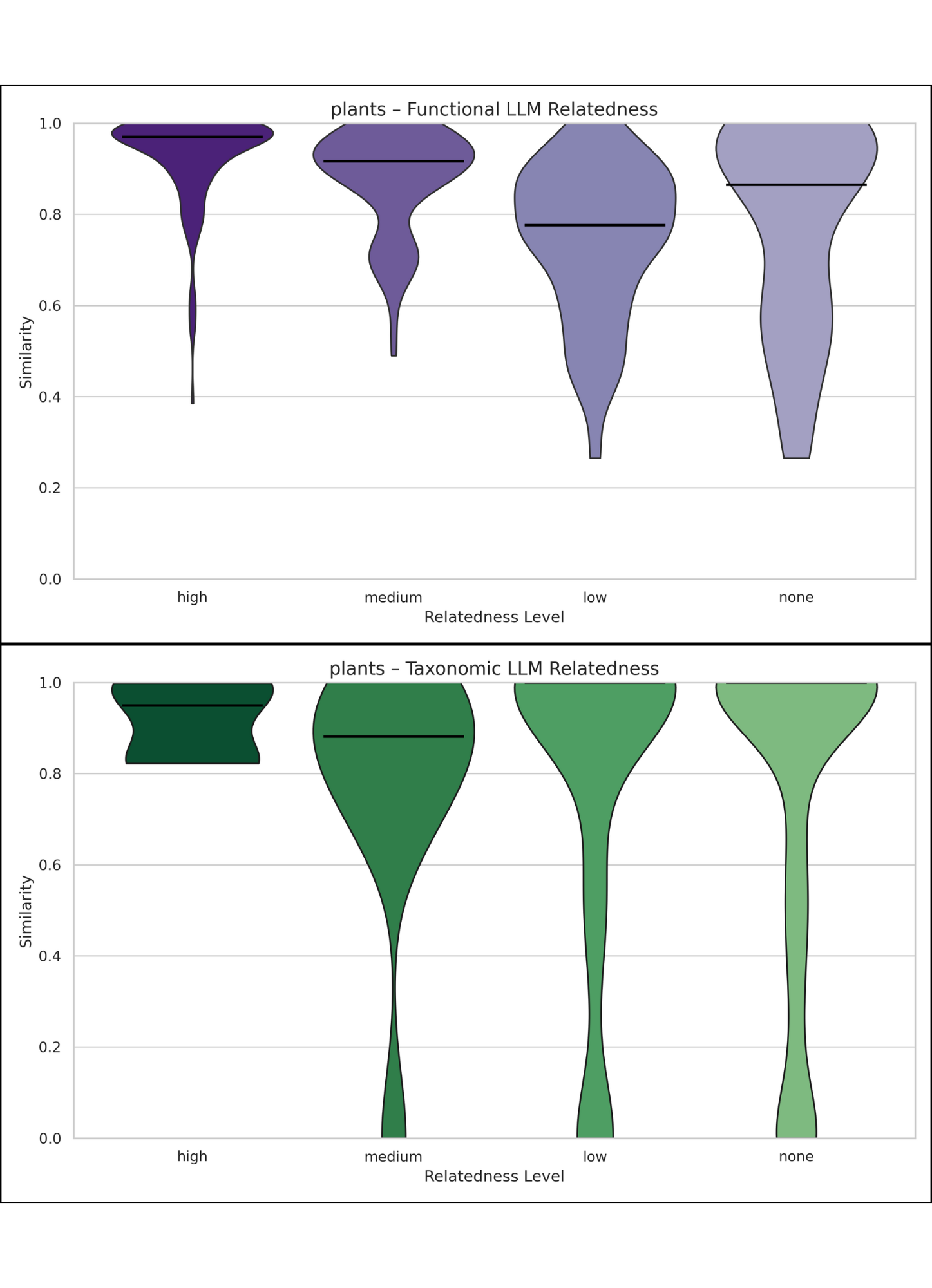


**Figure 12: Violin plots of functional (purple violin plots) or taxonomic (green violin plots) similarity for plant study pairs across biomes, grouped by LLM-assessed semantic relatedness.** The x-axis shows LLM-assessed semantic relatedness levels: high, medium, low, and none. The y-axis shows functional similarity values (range 0–1), where higher values indicate greater functional overlap. The width of each violin reflects the frequency of similarity values at a given level, and the horizontal bar indicates the median. **(a) Functional similarity scores for plant studies across LLM-assessed relatedness levels (purple violins).** Higher functional relatedness corresponded to higher and more consistent similarity scores, with the high condition showing a narrow, dense distribution near the upper limit and progressively broader, more variable distributions as relatedness decreased. The low and none levels exhibited wide ranges of similarity values, suggesting occasional functional overlap despite low semantic similarity. **(b) Taxonomic similarity scores for plant studies across LLM-assessed relatedness levels (green violins).** The high taxonomic relatedness group also showed tightly clustered high similarity values, but the medium, low, and none levels displayed broad overlapping distributions extending toward zero, suggesting weaker differentiation among these categories. Overall, functional relatedness appears to predict similarity more reliably than taxonomic relatedness, which only clearly separates highly related items while failing to distinguish lower relatedness levels.


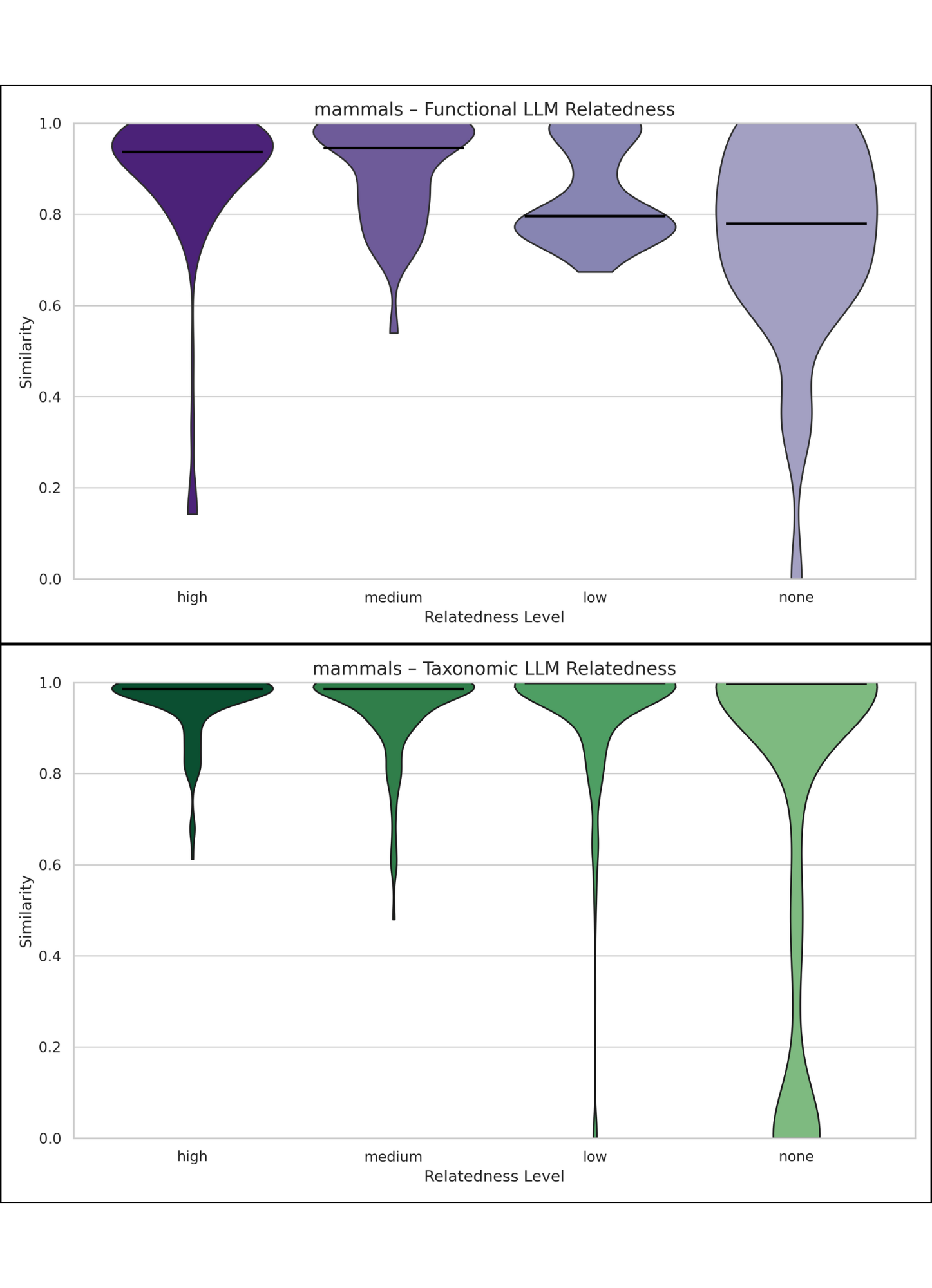


**Figure 13: Violin plots of functional (purple violin plots) or taxonomic (green violin plots) similarity for mammal study pairs across biomes, grouped by LLM-assessed semantic relatedness.** The x-axis shows LLM-assessed semantic relatedness levels: high, medium, low, and none. The y-axis shows functional similarity values (range 0–1), where higher values indicate greater functional overlap. The width of each violin reflects the frequency of similarity values at a given level, and the horizontal bar indicates the median. **(a) Functional similarity scores for mammal studies across LLM-assessed relatedness levels (purple violins).** While the high and medium functional relatedness groups show similarity scores concentrated near the upper range, their distributions are broader and more variable than in plants, including long tails toward lower similarity. The low and none levels likewise span wide ranges with substantial density in the higher similarity region, indicating that even semantically unrelated mammal studies can exhibit notable functional similarity. **(b) Taxonomic similarity scores for mammal studies across LLM-assessed relatedness levels (green violins).** All four taxonomic groups display strong density near maximum similarity, with broad overlapping distributions extending downward, particularly in the low and none conditions. This pattern suggests minimal taxonomic separation based on semantic relatedness categories, as taxonomic overlap remains prevalent regardless of LLM-estimated similarity. Overall, unlike plants, mammal similarity patterns reveal weaker functional and taxonomic discrimination across relatedness levels, reflecting greater structural similarity within mammal research independent of semantic relatedness assignments.

## 4. MGnify version-pipeline description

Each pipeline includes several key upgrades that focus on enhancing accuracy, flexibility, and compatibility with evolving technologies. Early versions (v1.0, v2.0, v3.0) include updated tools and databases, enhanced functional annotation, introduced RNA masking, and improved quality control. Pipeline v4.0 imported a major methodological shift by replacing the QIIME taxonomic classification component with MAPseq, while the Greengenes reference database was replaced with SILVA SSU / LSU version 128, enabling eukaryote classification. Version 4.1 further refined the key changes found in v4.0 by upgrading tools, such as SeqPrep and MAPseq, improving database quality by upgrading to SILVA 132, adding support for taxonomic assignments output in HDF5 file format, and making fixes to gene prediction coding. Lastly, version 5.0 represented a significant architectural process by introducing modularized data-specific workflows (for amplicons, raw metagenomic/metatranscriptomic reads, and assembly), defined in Common Workflow Language (CWL), ensuring reproducibility and transparency [[32]](https://www.zotero.org/google-docs/?8J06ty).

## 5. Excluded abstract text

The following publications have been found to be associated with 16 or more studies of the MGnify study dataset. Their abstract text has been found to be non-study-specific and has been excluded from the LLM relatedness analysis:

1. Mitchell AL, *et al*. EBI Metagenomics in 2017: enriching the analysis of microbial communities, from sequence reads to assemblies. Nucleic Acids Res. 2018 Jan 4;46(D1):D726-D735. doi: 10.1093/nar/gkx967. PMID: 29069476
2. Weißbecker C, *et al*. Dadasnake, a Snakemake implementation of DADA2 to process amplicon sequencing data for microbial ecology. Gigascience. 2020 Nov 30;9(12):giaa135. doi: 10.1093/gigascience/giaa135. PMID: 33252655
3. Mitchell AL, *et al*. MGnify: the microbiome analysis resource in 2020. Nucleic Acids Res. 2020 Jan 8;48(D1):D570-D578. doi: 10.1093/nar/gkz1035. PMID: 31696235
4. Větrovský T, *et al.* GlobalFungi, a global database of fungal occurrences from high-throughput-sequencing metabarcoding studies. Sci Data. 2020 Jul 13;7(1):228. doi: 10.1038/s41597-020-0567-7. Erratum in: Sci Data. 2020 Sep 15;7(1):308. PMID: 32661237

## 6. LLM full prompt

"How high similarity do you expect between these two microbiome studies? Instructions: focus on the biomes from which the samples were collected. In the next line quantify the similarity strictly according to these categories: '***high***' (studies of very similar biomes from the same organisms, closely related environments, and similar conditions), '***medium***' (studies of similar biomes from the same organisms or similar environments), '***low***' (studies of the same type of biome but from different organisms or environments) or '***no***' (completely unrelated studies, such as a host microbiome and a soil microbiome).\n\nStudy 1: ",

study_txt1,

"\n\nStudy 2: ",

study_txt2,

"\n\nPlease provide your response in JSON format with the following structure:\n{{\n \"explanation\": \"<short explanation>\",\n \"answer\": \"<***high*** or ***medium*** or ***low*** or ***no***>\"\n}}\nOnly return a valid JSON object."

## 7. Validation of LLM-generated labels versus manually-assigned labels

To evaluate the performance of the LLM, we manually curated a set of 175 randomly selected study pairs from a corpus of 2,901 unique studies. This subset was designed as a diagnostic validation sample to characterize agreement patterns and failure modes, rather than to provide a high-confidence estimate of final achievable performance. For each pair, we retrieved the LLM-assigned labels across the three runs and independently assigned a label based on human inspection. To summarize the LLM predictions, we determined the most frequent label across the three runs, producing a single consensus label per study pair. We then calculated the agreement frequency between the consensus LLM labels and the manually assigned labels for each category.

To provide a more comprehensive visualization of the results, capturing the distance from the correct answer, we generated a confusion matrix shown in Figure 13 below. Rather than collapsing performance into a single accuracy value, the confusion matrix reveals how the LLM’s misclassifications deviate from the correct category based on the human-curated label assigned. Most of the classifications are either on or close to the diagonal, with 94% (165 of 175) being at most one step away from the diagonal. Additionally, as the matrix illustrates, most discrepancies occur between neighbouring classes (close to the diagonal), demonstrating that even when the LLM is incorrect, its predictions are typically close to the manual judgment.


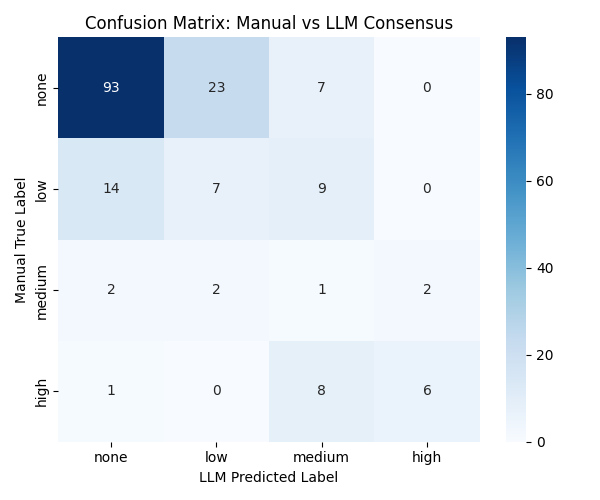


**Figure 14:** Confusion matrix comparing manually assigned labels with the LLM-consensus classifications for 175 randomly selected study pairs.

Given the substantial class imbalance in the manually curated dataset (70% of instances labeled as “None”), overall accuracy is not an appropriate summary metric. We therefore report per-class precision, recall, and F1-scores, as well as macro- and weighted-averaged F1 metrics (Table S2).

| **Category** | **Precision** | **Recall** | **F1-score** | **Support** |
| --- | --- | --- | --- | --- |
| None | 0.85 | 0.76 | 0.80 | 123 |
| Low | 0.22 | 0.23 | 0.23 | 30 |
| Medium | 0.04 | 0.14 | 0.06 | 7 |
| High | 0.75 | 0.40 | 0.52 | 15 |
| Accuracy |  |  | 0.61 | 175 |
| Macro avg | 0.46 | 0.38 | 0.40 | 175 |
| Weighted avg | 0.70 | 0.61 | 0.65 | 175 |

Table S2: Per-class precision, recall, and F1-score for the 175-pair manually curated validation subset.

The macro-averaged F1 score of 0.40 reflects the difficulty of the task across all classes, with performance deteriorating for the rarest categories. This pattern is expected in imbalanced classification settings, as achieving high precision and recall becomes increasingly difficult for infrequent labels. In contrast, the weighted average F1 score of 0.65 is dominated by performance on the majority “None” class, for which the model performs strongly (F1 score = 0.80), indicating that the LLM reliably identifies the absence of a relationship.

Notably, the qualitative pattern observed in the confusion matrix - namely that most errors occur between adjacent ordinal categories - remains consistent despite limited sample size, suggesting that the observed behavior reflects systematic model tendencies, rather than noise in the validation subset. As manual curation is expanded, particularly for underrepresented categories, these estimates are expected to stabilize, enabling more reliable imbalance-aware evaluation and potential improvements through rebalancing or ordinal-aware modeling strategies.

## 8. Acquisition of MGnify data via API

The dataset used in this project consists of taxonomic and functional profiles available on MGnify (https://www.ebi.ac.uk/metagenomics). To retrieve this data, we developed a “downloader” module in Python v3.5 that automates the acquisition of all files associated with each MGnify study. The module queries the MGnify API, collects all study information (in JSON format), extracts the corresponding study IDs, and creates a structured directory for each study on a local system.

For each study, the script navigates to the API's downloads section and retrieves all associated files, storing them in the study-specific directories specified. The process is parallelized using multiple threads to improve efficiency and includes error handling with automatic retries for failed downloads. After all available files per study have been downloaded, we subsequently selected taxonomic and functional profiles from versions 4.1 and 5.0 for the analyses, since older versions rely on outdated reference databases and legacy processing tools, which may introduce inconsistencies and reduce compatibility with more recent analyses.

The MGnify API provides a convenient JSON-based interface for exploring the available metadata and download endpoints. Examples follow:

- Available MGnify Studies List: <https://www.ebi.ac.uk/metagenomics/api/v1/studies>
- Specific Study’s Downloads List (e.g. MGYS00006855): <https://www.ebi.ac.uk/metagenomics/api/v1/studies/MGYS00006855/downloads>
- GO abundances file (functional profile) for study ID MGYS00006855: <https://www.ebi.ac.uk/metagenomics/api/v1/studies/MGYS00006855/pipelines/5.0/file/ERP135226_GO_abundances_v5.0.tsv>
- Taxonomic abundance file (taxonomic profile) for study ID MGYS00006855: <https://www.ebi.ac.uk/metagenomics/api/v1/studies/MGYS00006855/pipelines/5.0/file/ERP135226_taxonomy_abundances_SSU_v5.0.tsv>
